# Comparative performance of between-population vaccine allocation strategies with applications for emerging pandemics

**DOI:** 10.1016/j.vaccine.2022.12.053

**Published:** 2023-01-23

**Authors:** Keya Joshi, Eva Rumpler, Lee Kennedy-Shaffer, Rafia Bosan, Marc Lipsitch

**Affiliations:** a Center for Communicable Disease Dynamics, Department of Epidemiology, Harvard TH Chan School of Public Health, 02115 Boston, MA, USA; b Department of Mathematics & Statistics, Vassar College, 12604 Poughkeepsie, NY, USA

**Keywords:** Vaccine allocation, Vaccine distribution, Emerging pathogen, Pandemics

## Abstract

Vaccine allocation decisions during emerging pandemics have proven to be challenging due to competing ethical, practical, and political considerations. Complicating decision making, policy makers need to consider vaccine allocation strategies that balance needs both within and between populations. When vaccine stockpiles are limited, doses should be allocated in locations to maximize their impact. Using a susceptible-exposed-infectious-recovered (SEIR) model we examine optimal vaccine allocation decisions across two populations considering the impact of characteristics of the population (e.g., size, underlying immunity, heterogeneous risk structure, interaction), vaccine (e.g., vaccine efficacy), pathogen (e.g., transmissibility), and delivery (e.g., varying speed and timing of rollout). Across a wide range of characteristics considered, we find that vaccine allocation proportional to population size (i.e., pro-rata allocation) performs either better or comparably to nonproportional allocation strategies in minimizing the cumulative number of infections. These results may argue in favor of sharing of vaccines between locations in the context of an epidemic caused by an emerging pathogen, where many epidemiologic characteristics may not be known.

## 1. Introduction

The 2009 H1N1 influenza and the 2019 SARS-CoV-2 pandemics have highlighted the need for control and mitigation measures against emerging pathogens. SARS-CoV-2 has caused considerable morbidity and mortality, resulting in over 646 million cases and 6.6 million deaths worldwide as of December 2022 [1]. Previously, the H1N1 influenza pandemic was estimated to have caused around 200 thousand deaths in the first year globally [2,3]. Vaccines are currently the most effective public health intervention available against emerging pathogens and have greatly reduced severe disease outcomes [4].

Even with the approval and availability of vaccines, both pandemics have highlighted key challenges in the roll-out and uptake of vaccines globally during an ongoing epidemic. During the H1N1 influenza pandemic, global influenza A vaccine supply was much lower than initially estimated, resulting in large inequities in vaccine access across countries [5,6]. In its aftermath, the World Health Organization (WHO) developed a preparedness framework for the sharing of vaccines in a timely manner and encouraged advanced agreements for vaccine allocation and delivery [7,8].

The COVID-19 pandemic has seen similar challenges with initial vaccine supply falling far short of demand, slow vaccine roll-out, and highly unequal availability of vaccines across countries [9,10]. At the end of 2021, some countries had already vaccinated over 90 % of their population, while others did not have access to vaccines [9]. Overall, at the beginning of emerging pandemics, vaccination allocation decisions need to balance multiple factors in order to maximize the effect of each dose both within and across populations.

Across both pandemics, numerous papers have shown targeting specific subgroups within the population can result in decreased disease-related morbidity and mortality. For 2009 H1N1 influenza, models found prioritizing individuals at highest risk of complications resulted in the lowest morbidity and mortality [11–13]. For SARS-CoV-2, previous work [14–16] has shown, targeting specific subgroups within a given population—including older individuals—results in decreased morbidity and mortality. Other papers [17,18] found prioritizing certain occupational groups— including healthcare workers, and other essential workers—also decreased COVID-19 morbidity and mortality. However, previous theoretical work [19] has also shown that unequal vaccine allocations might be favorable in emerging infectious disease settings, but are less optimal when incorporating realistic assumptions about population heterogeneity and contact structure. This leaves a potentially conflicting message for policy makers when considering optimal allocation strategies.

We build upon this body of literature, by not only considering the optimal vaccine allocation, but also how the optimal allocation compares to all possible allocations across two populations. We illustrate these tradeoffs using a simplified model of an emerging pathogen similar to H1N1 or SARS-CoV-2 with a limited vaccine stockpile, varying the proportion of vaccines distributed between the two populations. We define the optimal vaccine allocation strategy as the one that minimizes the total number of infections across the two populations. Throughout, we compare vaccine allocations for multiple populations that distribute vaccines proportional to population size (pro-rata allocation) to those that distribute vaccines disproportionately, favoring either population (nonproportional allocations). Our results show that the efficiency gains for nonproportional allocations that are found in models with highly simplified epidemics are typically small; moreover, they vanish and can even reverse under settings more relevant to pandemics caused by emerging pathogens. We show that in more realistic scenarios incorporating a range of population, pathogens, vaccine and delivery characteristics, pro-rata allocation performs comparably to or considerably better than nonproportional distributions.

## 2. Methods

We use a deterministic, two-population, susceptible-exposed-infectious-recovered (SEIR) compartmental model (Fig. S21). We assume people are initially susceptible (S) and move to the exposed state (E) after an effective contact with an infectious individual. After a latent period, exposed individuals become infectious (I). After the infectious period has elapsed, infectious individuals move to a recovered state (R). We do not account for waning immunity and assume once individuals have recovered, they stay immune to infection for the duration of our simulation, here modeled as three years. We start by assuming that there is no interaction between the two populations, so all disease transitions happen in parallel between the two populations. The SEIR model equations are in Appendix A.2.1.

We extend this SEIR model to allow for underlying immunity (Figs. 3, S3, S4, S18) and vaccination (all Figures). At the start of the epidemic, in each population, individuals can be in the susceptible (S), infectious (I), or recovered (R) compartments. When there is underlying immunity, a set proportion of individuals are placed in R. Individuals in R, whether through infection or underlying immunity, can never be re-infected. When vaccines are distributed to the population, vaccinated individuals are placed in R if the vaccination is successful. We assume an all-or-nothing vaccine with 95 % efficacy, meaning 95 % of those who are vaccinated are placed in R and the remainder stay in S. When there is underlying immunity and vaccination, immune individuals may be vaccinated; vaccination has no effect on them, and they remain in R. Finally, we initialize each simulation by placing 0.1 % of individuals in I and allow the epidemic to run, unmitigated except by vaccination, through each population. Full model parameters and equations are shown in Appendix A.2. Where possible, parameters represent estimates from both the H1N1 influenza pandemic and the SARS-CoV-2 pandemic.

To recreate and extend on the results of Keeling and Shattock [19] we model two homogeneous populations with identical characteristics, except that population 2 has double the size of population 1 (Fig. 2). For later scenarios, which consider the impact of heterogeneity within populations, we simulate two populations that are identical in size, but vary in their population characteristics (e.g., fraction high risk) (Fig. 5, S8–S16).

First, within each population, we model efficient transmitters of infection, for example young adults or children [20,21] (Figs. 5, S8, S9, S11, S13, S15). We assume high transmitters are four times more likely to transmit compared to low transmitters [22]. We fix the within-population structure to allow the global *R*_0_ to equal 2, 4, 8 or 16. SEIR model equations are in Appendix A.2.4 and the full *R*_0_ derivation is shown in Appendix A.2.6.

Second, we instead model individuals at elevated risk of death from infection (Figs. 5, S8, S10, S12, S14, S16). For COVID-19 this represents elderly individuals or individuals with co-morbidities known to exacerbate disease [23,24]. For the H1N1 influenza pandemic, this represents young adults. We assume these individuals are five times more likely to die than other infected individuals [11,25,26]. The SEIR model equations are in Appendix A.2.5.

In both high-risk scenarios, we initialize the number of susceptible and recovered individuals with the total number of vaccine doses split amongst the two populations according to the allocation. Within each population, high-risk individuals are vaccinated first, with leftover doses then allocated to the low-risk population, as described in Appendix A.2.7.

Finally, we model the scenario where vaccines are unavailable at the start, but are progressively rolled out over the course of the epidemic (Figs. 4, S5–S7, S9–S16, S20). Here, we vary both the timing of roll-out (1, 10, 30, 50, or 100 days after the epidemic has begun), and the fraction of the population vaccinated each day (1 % to 3 % per day).

For each simulation we calculate the cumulative number of infections and deaths from the deterministic SEIR model at the end of the epidemic. In most scenarios, we define the optimal allocation strategy as the one that minimizes the total epidemic size (cumulative number of infections) across both populations. Within the high morbidity scenario, however, we define the optimal allocation strategy as the one that minimizes the total number of deaths across both populations. This is equivalent to maximizing the total number of people across both populations that escape infection (or death) [27].

**Fig. 1.**
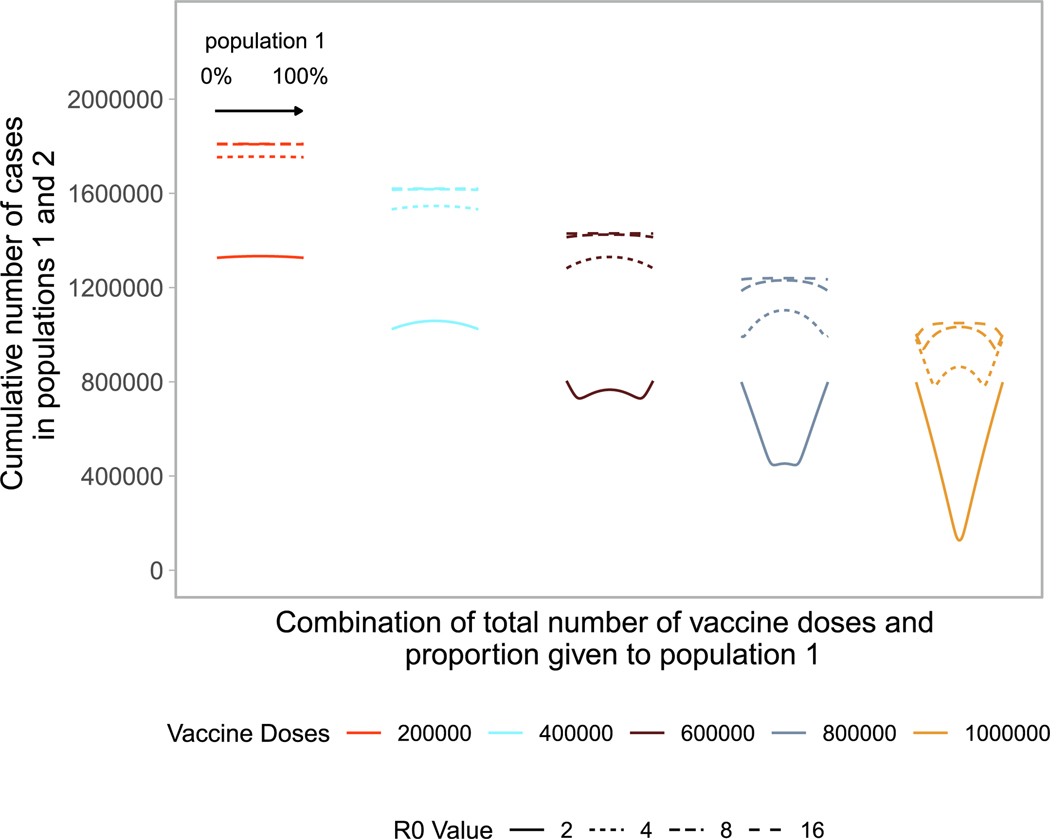
Performance of different allocation strategies of a limited vaccine stockpile across two homogeneous population of equal size with no underlying immunity and prophylactic vaccination. Both populations have one million individuals. Each color represents a different number of total vaccine doses. Each line represents a different basic reproductive number between 2 and 16. Each curve shows the cumulative number of cases across both population 1 and 2 for different proportions of doses given each population. Across each curve, from left to right, the proportion of doses to population 1 goes from 0 to 100%. Conversely, for population 2, the proportion of doses goes from 100% to 0%.

We conduct sensitivity analyses to assess the robustness of our results. For each of the scenarios presented we additionally consider the impact of varying *R*_0_ between 2 and 16, allowing for improved understanding across a variety of pathogens or viral variants [28] (Figs 1, 3–5, S1, S3–S16, S20). Next, we model a leaky vaccine scenario where we assume the vaccine reduces susceptibility to infection for each individual by 95 % (Fig. S1). As a result, all vaccinated individuals (except those previously immune through natural infection) can become infected with the virus, although the probability of infection for each contact with an infected individual is lower than for an unvaccinated individual. Next, for the scenario where populations have unequal sizes, we allow the size of population 2 to be ten times the size of the other, to show how allocation decisions perform in a more extreme case. (Fig. S2). We additionally model scenarios where there is a very large percentage of the population immune from prior infection, varying levels between 50 and 80 % (Fig. S3) and allowing levels of underlying immunity to be different across the two populations (Fig. S4). We further extend the model by allowing the timing and speed of roll-out to vary between the two homogeneous populations (Fig. S5–S7). For heterogeneous populations, we consider continuous roll-out scenarios with 25 % and 50 % of each population considered high-risk of either transmission (Fig. S9, S11) or mortality (Fig. S10, S12), and allow timing and speed of roll-out to vary between the two populations (Fig. S13–S16). We relax the assumption of 95 % vaccine efficacy by modelling a range of values from 50 % to 90 % (Fig. S17) across different levels of underlying population immunity (Fig. S18). For all scenarios thus far, we have considered a highly transmissible pathogen with very high R_0_ values. We model a scenario where the R_0_ of the pathogen is between 1.2 and 2 (Fig. S19). Finally, we further extend the model by relaxing the assumption that the two populations do not interact (Fig. S20). We allow a fraction i of infected individuals in both populations to contribute to the force of infection in the other population instead of their own population. An *i* value of 0 corresponds to no interaction, and an *i* value of 0.5 corresponds to complete interaction between the two populations (i.e,. is equivalent to one large population). Table S1 shows the full list of analyses considered. All analyses were conducted in R version 4.2.1.

## 3. Results

For each section below, in scenarios where the population size is identical across the two populations, pro-rata allocation is equivalent to equal allocation across populations and nonproportional allocation refers to one population receiving more doses than the other. For scenarios where one population is larger than the other, pro-rata allocation refers to allocation proportional to population size, and nonproportional when allocation is not proportional to population size.

### 3.1. Literature review

We reviewed the literature on optimal vaccine allocation across populations that was published prior to the emergence of SARS-CoV-2 (Appendix A.3.1). Multiple papers [19,29–32] have shown that allocation proportional to population size (i.e., pro-rata allocation) is rarely optimal. Further, previous studies have highlighted that the timing of vaccine allocation [33–35], heterogeneity in population composition, as well as the stochasticity in infection dynamics affect the optimal distribution [36,37]. Duijzer et al. [27] provide important contributions by showing that the optimal vaccination threshold is often less than the herd immunity threshold as further detailed in Appendix A.3.2.

### 3.2. Optimal allocation in two populations of equal size

We build upon the existing literature by first examining allocation decisions in the simple scenario of two identical, non-interacting populations with no underlying immune protection to the pathogen (Fig. 1). In the simplest case, with a small number of vaccine doses available, pro-rata allocation performs comparably to highly nonproportional allocation strategies (e.g., flat line in Fig. 1). As the number of vaccine doses increases, highly nonproportional strategies gain advantage over pro-rata allocation (e.g., upside down ‘‘U” shape). This occurs because one population can be vaccinated close to, but lower than, its herd immunity threshold, maximizing the indirect effect of the vaccine doses [27]. When sufficient vaccines are available for both populations to reach that threshold, more nonproportional strategies use the doses less efficiently, (e.g., increasing arms of the ‘‘W” shape). Allocating doses to the population that has reached its threshold provides limited benefit in that population and withholds doses from the other. When there are nearly enough doses to reach the thresholds in both populations, the optimal strategy becomes pro-rata allocation between the two populations (e.g., ‘‘V” shape).

As the basic reproductive number increases, we again see that nonproportional allocations perform optimally, as the number of available doses is less than the number needed to reach the critical herd immunity threshold in both populations. In these scenarios, vaccinating one or the other population up to its critical herd immunity threshold results in the lowest cumulative cases across both populations. At very high basic reproductive numbers (i.e., *R*_0_ = 16), pro-rata allocation performs comparably to highly nonproportional allocation strategies, as few individuals can be protected indirectly even with widespread vaccination. If the reproductive number is unknown or estimated incorrectly, nonproportional allocations may be highly sub-optimal.

### 3.3. Optimal allocation in two populations of unequal size

Extending the simple case of non-interacting populations of equal size, previous studies have shown how optimal allocation across populations of different sizes is not linear, but varies with the number of doses available in a characteristic, and often counter-intuitive, ‘‘switching” pattern [19,27,30].

**Fig. 2.**
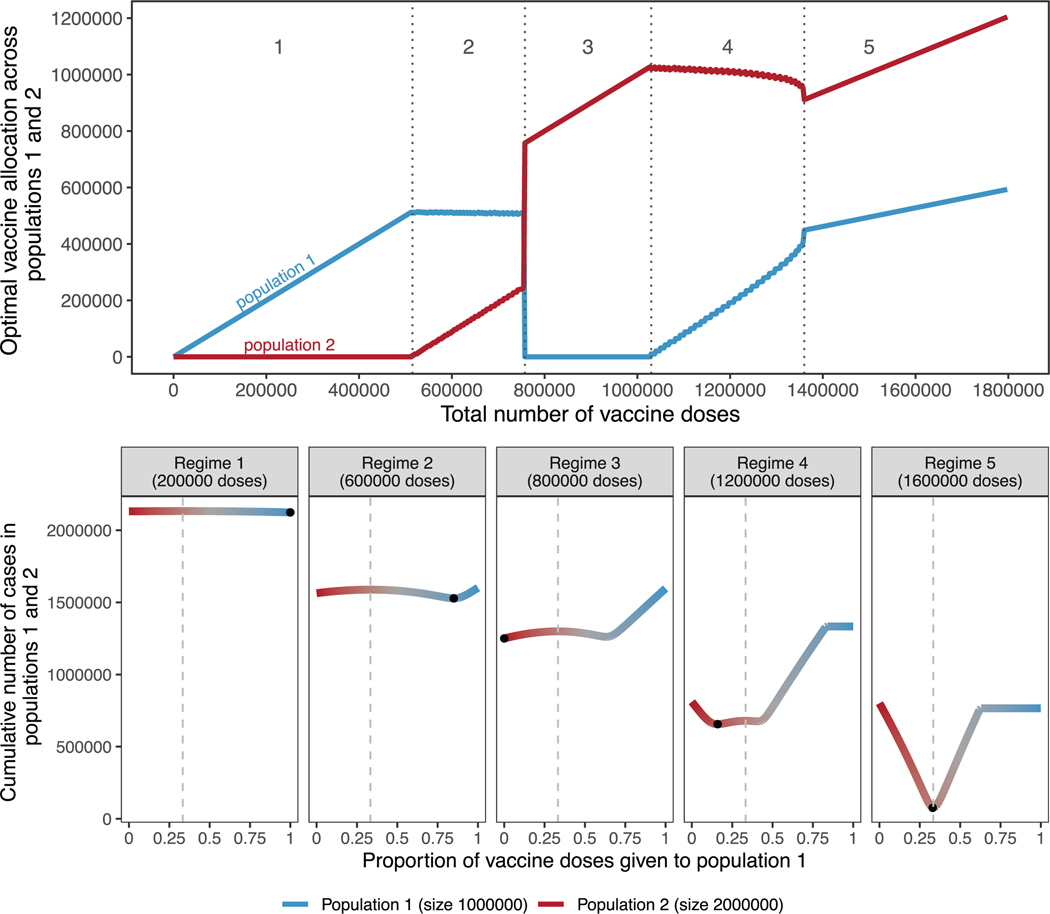
Top: Optimal allocation strategies of a limited vaccine stockpile across two homogeneous populations of unequal size with no underlying immunity, prophylactic vaccination and an R_0_ of 2. Populations 1 (blue) and 2 (red) have one and two million individuals, respectively. Dotted vertical lines were added to highlight regimes (1 to 5) showing different vaccine allocation patterns. Bottom: Performance of allocation strategies for five different numbers of vaccine doses, representative of the regimes shown in the top half of the Figure. Color coding corresponds to vaccine allocation ranging from giving all doses to population 2 (red) to giving all doses to population 1 (blue). The optimal allocation, the minimal value on each plot, is highlighted by a black point. Dashed vertical lines in the bottom panel represent pro-rata allocation between population 1 and 2. (For interpretation of the references to color in this figure legend, the reader is referred to the web version of this article.)

As shown in Fig. 2 (top), when the number of doses available is very limited, optimal allocation concentrates all vaccine doses to the smallest population, not assigning any to the largest population (regime 1). As the number of doses allocated to the smaller population reaches its threshold, additional doses are gradually allocated to the larger population (regime 2). Strikingly, a switch happens between regimes 2 and 3, and in regime 3 all doses are allocated to the larger population and none to the smaller one. Then, as the largest population itself reaches its threshold, supplementary doses are assigned to the smaller population (regime 4). When the number of vaccines available allows both populations to attain their respective thresholds, vaccines are allocated proportionally to the population sizes (regime 5).

For most values of vaccine available, the optimal allocation is highly nonproportional (regime 1, 2, 3, 4), as previously shown [19,27]. This counterintuitive result is caused by the nonlinearity of the indirect effect from each additional vaccine dose. Additional doses are allocated to the population where they have the largest benefit. For example, in regime 1 of Fig. 2, additional doses bring a larger benefit in the smaller population then they would in the larger population.

Importantly, while prior literature [19] demonstrates that nonproportional allocations can be optimal, these results show that the benefit of such nonproportional optimal allocations over more pro-rata ones is often small. As shown in Fig. 2 (bottom), for low numbers of vaccine doses (regimes 1 and 2), although concentrating all doses to the smallest population is optimal, other strategies do not perform much worse. Each regime is characterized by a different allocation profile that gives rise to a different optimum, indicated by black points. In regime 4, the characteristic W shape appears where a fully nonproportional allocation is sub-optimal, regardless of which population is vaccinated. We see similar results when population 2 is ten times the size of population 1 (Fig. S2).

### 3.4. Impact of underlying immunity

As vaccines become available to different locations at different points in their local epidemic, populations will have varying degrees of underlying immunity to the virus due to prior infections. Serological surveys estimate that at the end of 2020, around a fifth of the population had already been infected in areas hardest hit during the spring of 2020 [38,39]. More recent estimates show seroprevalence increased to almost 60 % after the Omicron variant became predominant in the United States [40]. Select high-risk groups, including health care workers and nursing home residents, have been shown to have an even higher prevalence of SARS-CoV-2 antibodies [41]. To account for underlying immunity, we further simulate optimal allocation decisions with varying levels of underlying immunity in each population to mirror the fact that allocation decisions are made during an ongoing pandemic.

**Fig. 3.**
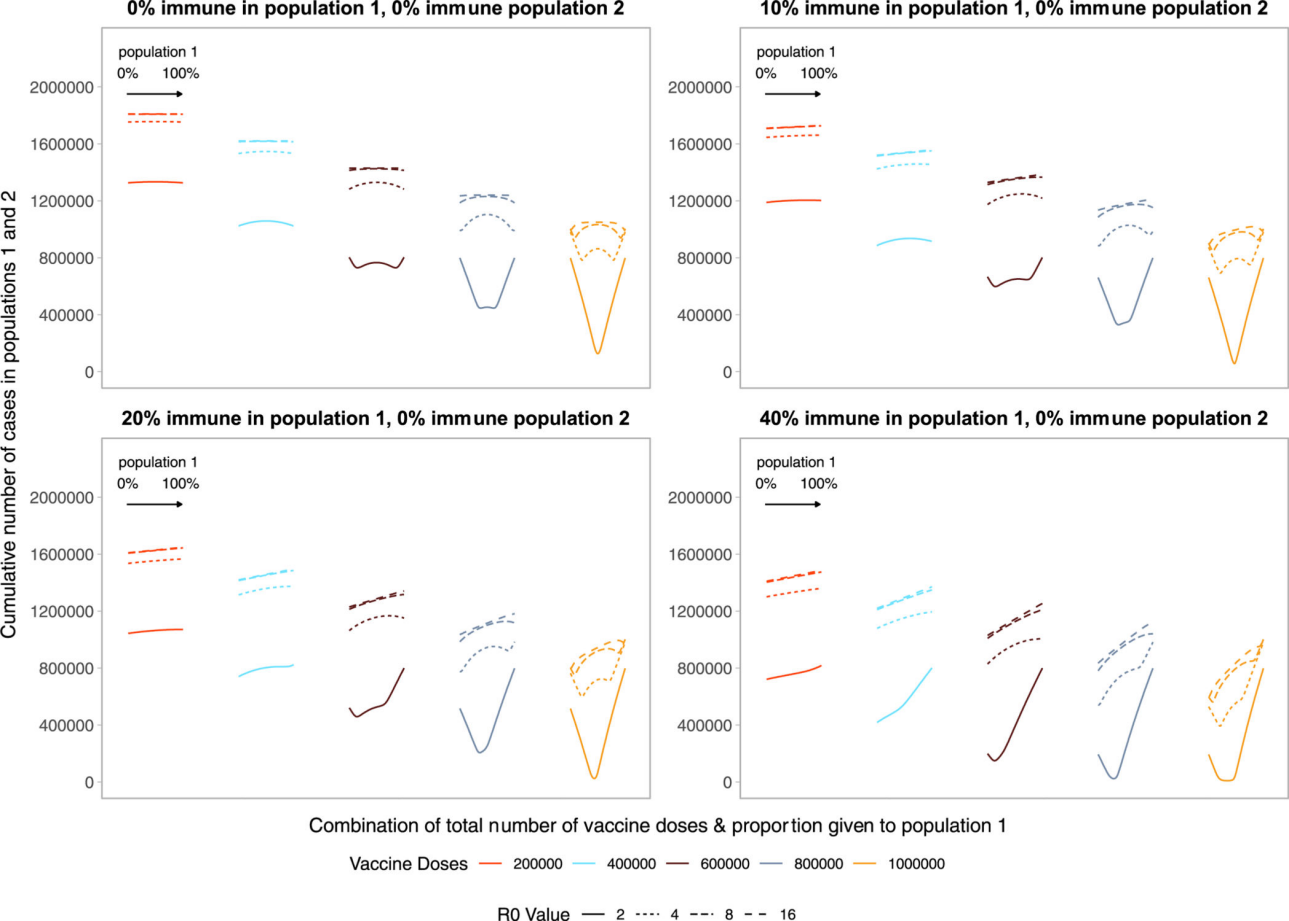
Performance of different allocation strategies of a limited vaccine stockpile across two homogeneous populations of equal size (one million individuals) with different underlying immunity, and prophylactic vaccination. We fix population 2 to have no underlying pathogen immunity and vary underlying immunity in population 1 from 0 to 40%. Each color represents a different number of total vaccine doses. Each line represents a different basic reproductive number between 2 and 16. The panel on the top left is equivalent to Fig. 1.

Comparing two populations with varying amounts of underlying immunity, the optimal strategy favors prioritizing the population that is closer to their herd immunity threshold (Figs. 3, S3, S4, S18). Fig. 3 shows optimal allocation decisions across two homogeneous populations of equal size with no immunity (top left, repeating Fig. 1) or increasing degrees of immunity in population 1. With increasing immunity in population 1, the characteristic V- or W-shape becomes more lopsided as fewer doses are required in population 1 to reach the threshold at which doses should be split between populations. Extremely nonproportional allocation strategies either waste doses or fail to minimize the cumulative number of infections in both populations if given completely to population 1 or 2, respectively. In addition, allocating vaccines to population 1 beyond the amount needed to reach its threshold results in the highest cumulative number of cases because it confers little additional benefit in population 1, and deprives population 2 of vaccines needed to mitigate cases. Note that this makes the nonproportional approach risky, as the precise extent of prior immunity is unlikely to be known. As before, once the number of doses is large enough to approach or reach the threshold in both populations, optimal strategies move closer to pro-rata allocations. The same results hold as we increase the level of underlying immunity from 50 to 80 % in the population (Fig. S3). When populations vary in their level of immunity, the total number of doses wasted decreases as a larger total fraction of individuals across populations are immune and a larger combination of allocations result in getting both populations to their herd immunity threshold (Fig. S4). As we consider populations with some underlying immunity, we find that as the level of vaccine efficacy increases, allocation decisions quickly favor pro-rata distributions for low R_0_ values (e.g., R_0_ = 2) (Fig. S18).

As we vary the basic reproductive number, holding vaccine doses fixed, we find the characteristic ‘‘V” and ‘‘W” shapes are shifted to the left. The number of vaccine doses needed to reach the critical herd immunity threshold increases as the basic reproductive number increases. Nonproportional approaches become more favorable as the level of underlying immunity in population 1 increases, because fewer doses are required for population 1 to reach their herd immunity threshold. Thus, even for very high *R*_0_ values, the optimal strategy, minimizing the cumulative number of cases across both populations, prioritizes allocating doses to the population that is closest to reaching its critical herd immunity threshold.

### 3.5. Impact of delayed vaccine roll-out in a homogeneous population

**Fig. 4.**
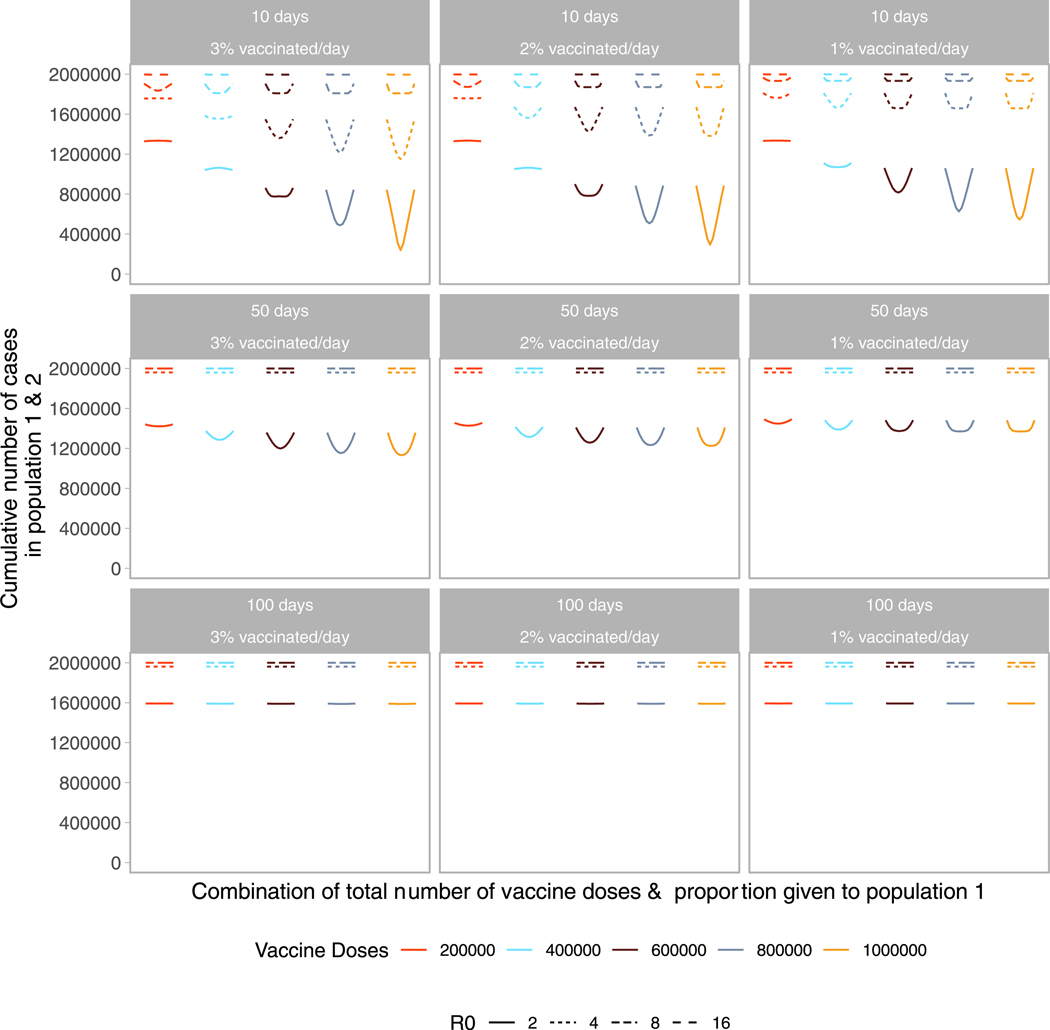
Performance of different allocation strategies of a limited vaccine stockpile across two homogeneous populations of equal size (one million individuals) with no underlying immunity, with vaccines rolled out at different speeds and different times after the start of the epidemic. Each color represents a different number of total vaccine doses. Each line represents a different basic reproductive number between 2 and 16. We vary both the timing and speed of roll-out between 10, 50 or 100 days after the start of the epidemic with 1, 2, or 3 % of the population vaccinated per day. Each column represents a given roll-out speed while each row represents a different timing.

Next, we examine the impact of vaccine roll-out over the course of the epidemic. We find both the timing and speed of roll-out play an important role in minimizing the final size of the epidemic. As shown in Fig. 4, the cumulative number of cases across both populations is minimized when vaccine roll-out occurs as soon as possible after the start of the epidemic. Further, the final size is minimized when roll-out speed is increased, vaccinating a larger proportion of the population each day.

For the early and efficient roll-out (beginning 10 days after the start of the epidemic, at a rate of 2 or 3 % of the population/day), the vaccination performance profile across possible allocations looks similar to that of the prophylactic vaccine deployment strategy shown in Fig. 1. However, for a slower or more delayed roll-out we see highly nonproportional approaches perform poorly across almost all doses and pro-rata approaches result in the smallest final size. This is because a larger fraction of the population is naturally infected, minimizing the gains from concentrating vaccine doses in one population.

As we incorporate differences in transmissibility, we find timing and speed to be of greater importance (Fig. 4, S5–S7). Even with a vaccine roll-out 50 days after the start of the epidemic, there are no differences in final size across all allocation strategies, within a given *R*_0_ level, as the epidemic has ended in the population before vaccines are introduced. For higher reproductive numbers, faster, earlier roll-outs are needed for vaccination to have an impact on the total number of infections. Note that the lower R_0_ scenarios may better represent settings where non-pharmaceutical interventions that reduce the effective reproductive number are in place until vaccination. In situations where the populations have differences in timing and speed, we find that the cumulative number of infections is minimized when vaccines are allocated to the population that has the earlier or faster roll-out (Fig. S5–S7).

### 3.6. Impact of heterogeneous population structure

Looking within a population, many studies have shown optimal strategies favor prioritizing older individuals (e.g., those aged 60 or over) or those with certain comorbidities when the goal is minimizing mortality. If the goal instead is minimizing final size, targeting adults 20–49 with an effective transmission-blocking vaccine minimizes cumulative incidence [14,15]. Here we model the impact of heterogeneous population structure to examine the impact of strategies across populations. These simulations consider populations with heterogeneous transmission or with heterogeneous risk of death.

**Fig. 5.**
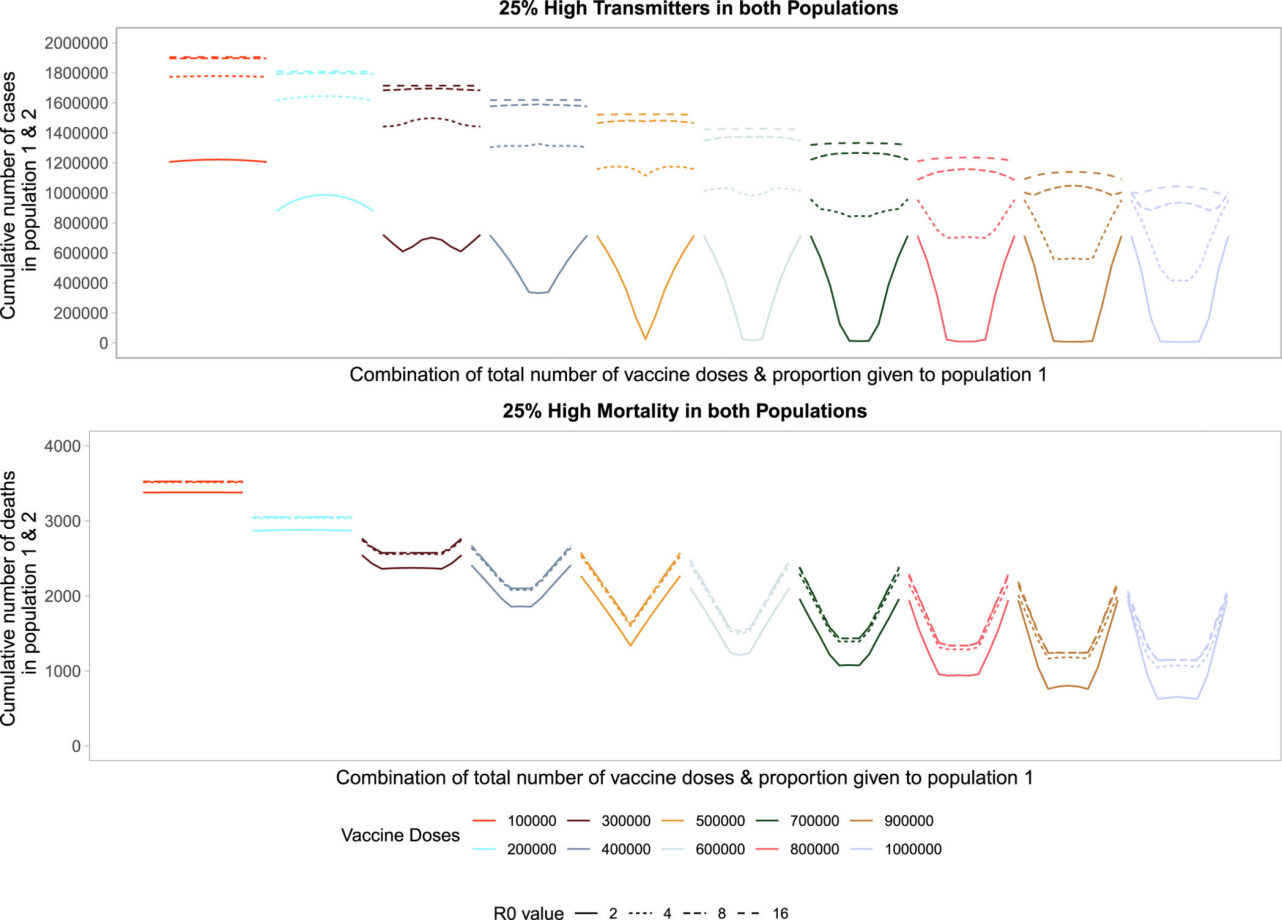
Performance of different allocation strategies of a limited vaccine stockpile across two heterogeneous populations of equal size (one million individuals) with no underlying immunity and prophylactic vaccination. Each color represents a different number of total vaccine doses. Each line represents a different basic reproductive number between 2 and 16. In both the high transmission scenario (top) and high mortality scenario (bottom), 25% of both populations are high risk.

Targeting high transmission or high mortality groups first within a population shifts the optimal allocation across the two populations towards pro-rata allocation (Fig. 5, S8). In Fig. 5 we first model the impact of prophylactic vaccination in a heterogeneous population structure with 25 % of each population at either high risk of transmission (top) or death (bottom). In the high-transmission scenario, the behavior looks similar to that in Fig. 1 for a low number of doses, representing the trade-off between vaccinating the high-transmitters in both populations. Once there are enough doses available to vaccinate enough high transmitters, to reduce transmission dramatically, the optimal strategy favors more pro-rata allocations across the two populations, since the herd immunity threshold has effectively been reached by eliminating the bulk of transmission risk. This shift closer to pro-rata allocations occurs at a lower number of vaccine doses compared to Fig. 1. In the high-mortality scenario, we see the optimal allocation rapidly shift to pro-rata strategies, starting at a very low number of vaccine doses. Interestingly, the sequence of profiles from Fig. 1 is repeated twice. First, for a low number of vaccine doses there is a trade-off between vaccinating the high-mortality individuals in both populations. Then for higher vaccine counts the trade-off is repeated, this time between all individuals of both populations. While this trade-off exists, pro-rata allocation is heavily favored across almost all levels of available vaccine doses.

Looking across different levels of *R*_0_, we find similar trends. Vaccinating higher transmission or mortality groups first results in more pro-rata allocation strategies across populations. For higher *R*_0_ values (i.e., 8 or 16) pro-rata allocation performs comparably to highly nonproportional strategies. Increasing the proportion of high-risk individuals to 50 % of the population (Fig. S8) we find similar trends for *R*_0_ values of 2 and 4. For higher *R*_0_ values, nonproportional approaches perform optimally as a larger fraction of the population is driving transmission, so effectively targeting this group in either population minimizes the cumulative number or deaths or cases across the two populations.

Next, we considered the impact of continuous roll-out for both the high transmission and high mortality scenarios. We find that across both high-risk scenarios and all vaccine roll-out times and speeds, nonproportional allocation is highly sub-optimal (Fig. S9–S12). Similarly to Fig. 4, we vary the start date of vaccination roll-out (1, 10, 30, 50, or 100 days), the daily vaccination rate (1, 2 or 3 % per day), and the proportion of the population at high risk (25 or 50 %). We find that both the speed and timing of vaccine roll-out are important factors in minimizing the cumulative number of cases or deaths across the two populations and see the greatest reduction in cumulative deaths and final size with the earliest and fastest roll-out. Specifically, for vaccine stockpiles larger than 500,000 doses, the achievable impact of vaccination is more dependent on the timing (solid vs dashed curves) and speed (different panels) of vaccine roll-out rather than on the total number of doses available. As we allow either the timing (Figs. S13, S14) or speed (Figs. S15, S16) to vary between the populations, we find that allocating doses to the population that has the earlier or faster roll-out minimizes the cumulative number of infections across both populations, similar to the homogeneous scenario.

### 3.7. Sensitivity analyses

We assessed the robustness of our results by varying the characteristics of the vaccine and connection between populations to be more representative of the current pandemic. As expected, the leaky and all-or-nothing vaccine have the same critical vaccination threshold, though the cumulative number of cases in the leaky vaccine scenario is equal to or larger than the all-or -nothing scenario [42] (Fig. S1).

As vaccine efficacy decreases from 90 % to 50 %, the cumulative number of cases across both populations increases, the critical herd immunity threshold increases and pro-rata allocation strategies become less favorable, with nonproportional allocations being optimal in some cases for the lowest efficacy values. Even for these situations, however, the advantage of nonproportional allocations is modest (Fig. S17). Next, as we increase immunity levels across both populations, the total number of cases is reduced, and the critical herd immunity threshold is lowered. Even with low vaccine efficacy values, in scenarios with high underlying immunity, the critical herd immunity threshold can be reached, and pro-rata allocations are favored (Fig. S18).

For situations where R_0_ is less than two, which might be more realistic for an emerging pathogen, we find pro-rata allocations quickly become optimal. For an R_0_ of 1.2, pro-rata allocation strategies are always optimal across the number of vaccine doses considered. As the R_0_ increases, pro-rata allocation becomes favored as the number of vaccine doses increases. For the largest number of vaccine doses, pro-rata allocation is the optimal strategy across all R_0_ values (Fig. S19).

In all previous situations we have only considered the scenario of non-interacting populations. As we relax this strict assumption, we find that as the amount of interaction between the two populations increases, pro-rata strategies are most favorable (Fig. S20). When the force of infection in each population depends on epidemic dynamics in both populations, accounting for interaction drastically changes the optimal allocation profiles and favors pro-rata allocation between populations, as seen in previous work [19,27]. Even for low values of the interaction parameter *i*, pro-rata allocation rapidly becomes optimal. As *i* increases, the difference in outcomes between nonproportional and the optimal pro-rata allocation strategies decrease, as indicated by the flattening of the curves. For i equal to 0.5, the two populations concretely behave like one large population. Compared to the non-interacting case, allowing for interaction between the two populations leads to a higher cumulative number of infections for all possible vaccine allocation strategies, and the ‘‘W”-shaped allocation curve no longer appears.

As we increase the basic reproductive number, we find that the optimal strategy quickly favors pro-rata allocation decisions. In addition, interaction between the two populations becomes less important for very high values of R_0_, as the allocation profiles look similar across all interaction parameters for R_0_ values of 8 and 16.

## 4. Discussion

In emerging pandemics, countries must make challenging vaccine allocation decisions despite imperfect knowledge about the epidemic, resource constraints, and even the availability and effectiveness of vaccines. Previous studies [19] have shown simple scenarios favor nonproportional allocation. We recreate those findings and further extend vaccine allocation theory, applying it to a wide range of scenarios, with disease parameters similar to the SARS-CoV-2 and H1N1 influenza pandemics. We focus on these two emerging pathogens because they provide the greatest amount of data and understanding, in part because vaccines were deployed while the pandemic was ongoing.

In the simple case of two non-interacting populations of identical size with homogeneous risk structure and without underlying immunity, we find that until there are enough vaccine doses for both populations to approach their critical herd immunity threshold, nonproportional strategies perform optimally. We highlight however, that in this situation pro-rata allocation performs either comparably or not considerably worse than highly nonproportional approaches. For a larger number of vaccine doses, pro-rata allocation strategies outperform more nonproportional approaches.

When considering populations of unequal sizes, we find similar results. For vaccine numbers smaller than the number required to bring both populations close to their herd immunity thresholds, the optimal allocation is nonproportional, concentrating all the doses in the largest population that can approach its herd immunity threshold, as supported by Keeling and Shattock [19]. Similarly to the equal population size scenario mentioned above, we find that the performance of pro-rata allocation is not considerably worse than the optimal one.

We consider several aspects of the diversity and complexities of emerging pandemics—including underlying immunity, asymmetric vaccine rollout, heterogeneous population structure, differences in underlying disease transmissibility, and the interaction between some of these parameters—to understand how these decisions may change in more realistic settings. We find that across a range of scenarios considered, optimal allocation decisions often favor pro-rata allocation across populations as complexity is added. Further, for situations where nonproportional strategies are optimal, pro-rata allocation strategies often perform comparably. Since these strategies are often optimal or nearly optimal across a range of parameters, while nonproportional allocations are only generally optimal for narrow parameter ranges, more pro-rata strategies might be the best option under uncertainty in an ongoing epidemic where many of these parameters are either unknown or changing over the course of the epidemic. In addition, determining the optimal amount of nonproportionality depends on detailed knowledge of all of these parameters, which will rarely, if ever, be available; in several cases a more extreme nonproportional allocation can be worse than the pro-rata allocation. Overall, these results supports the European Commission’s decision to allocate vaccine doses proportional to population size among the 27 European Nations [43].

Moreover, during an emerging pandemic, it may be unclear whether the newly developed vaccines confer protection against transmission, thus limiting the potential benefit from nonproportional vaccine allocation strategies that rely on maximizing the benefit from indirect protection in one population. It may thus be preferable to focus on pro-rata allocation strategies as those rely more on the direct protection against disease. Parameter values from COVID-19 and pandemic influenza illustrate this phenomenon; however, these results contribute more generally to the existing vaccine allocation theory for any epidemic emerging in multiple populations when key epidemic variables remain unknown.

For scenarios considering heterogeneous population risk, we find that when first targeting high risk individuals, either high-transmitters or those at higher risk of death if infected, allocations closer to pro-rata between populations is optimal. In scenarios considering continuous vaccine rollout within heterogenous populations, we find that across all levels of vaccine considered, pro-rata distribution either outperforms or performs comparably to nonproportional approaches. Targeting high-risk individuals first, then shifting priority to lower-risk individuals is supported by previous modeling work, looking at SARS-CoV-2 vaccine allocations within a single population [14–16,44,45]. The ongoing COVAX strategy follows this approach, as did the roll-out in the USA, which vaccinated health care workers and elderly individuals first [46,47]. This also supports previous guidelines for H1N1 influenza which first targeted vaccines to high-risk groups [48].

Our modeling analyses are subject to many simplifying assumptions on population dynamics and population and pathogen characteristics that could impact the generalizability of our results. First, we made modelling choices regarding vaccination. While we consider ranges of vaccine efficacy in a sensitivity analysis (50 %−90 %), most of our analyses were conducted with a fixed efficacy (95 %) that did not vary over time. We consider a vaccine that prevents both disease and infection, thus providing some indirect protection to the unvaccinated population. While some vaccines reduce infectiousness, in future pandemics this effect will still need to be precisely assessed. Next, we do not consider delays between doses or the time it takes to build immunity. The results here can be considered the scenario where immunity from the final dose occurs at the modeled time of vaccination. Further, we only model one available vaccine. The SARS-CoV-2 pandemic illustrates how the vaccine landscape can be complex, as multiple vaccines are available. Considerations for optimal allocation in this context are more complicated and trade-offs need to be weighed between vaccines with different characteristics, such as immunogenicity profiles, number of doses, and timing and/or speed of roll-out [49]. Additionally, we do not model vaccine refusal and assume that all individuals given doses accept them. Refusal of doses could lead to a lower overall impact of vaccination.

Second, we made assumptions regarding population characteristics. We model a heterogeneous population structure in a simplistic way, considering high risk of mortality and transmission separately, which may not adequately represent a true complex population structure. Furthermore, we only model allocation strategies within two symmetric populations. It is likely that policy makers will face allocation decisions across multiple countries, or across multiple regions within a country. While our analyses do not extend to more than two locations, general principles should remain the same, as illustrated elsewhere for three populations [19,27]. Additionally, except for the sensitivity analysis considering a leaky vaccine (Fig. S1), we did not allow reinfection (i.e., recovered but susceptible) of individuals that are recovered either from natural infection or from vaccination; if immunity wanes over time, then, the decision may get more complex. Finally, we do not consider the implementation of non-pharmaceutical interventions (NPIs) due to the difficulty in picking parameters that adequately represent their evolving implementation and adherence across time and location. This leads to epidemics in our model spreading through the population largely unmitigated.

Third, we chose to not focus on a specific pathogen, but varied R_0_ over a wide range of values between 2 and 16. As these larger R_0_ values might not be representative of a ‘‘typical” emerging pathogen, we additionally included a sensitivity analysis with lower R_0_ values between 1.2 and 2. We did not consider varying R_0_ over the course of the epidemic as could happen with emerging variants for example.

Future modeling work on vaccination strategies during emerging pandemics is needed, for example considering scenarios where multiple vaccine candidates are rolled out simultaneously. These studies should also consider multiple endpoints, including the effects of vaccines on reducing hospitalizations and preserving hospital capacity, which may have indirect benefits for mortality rates for COVID-19 and other diseases beyond the direct prevention of infection in high-risk populations. In addition, other work should also consider populations with varying epidemic dynamics, and distribution capacity. Indeed, it has been argued that populations at higher immediate risk of disease spread and populations where vaccine roll-out is most efficient should be prioritized for vaccine allocation [50]. Further, for our analyses we focus on the interplay between a few factors. We know epidemic dynamics for an emerging pathogen can be complex, and future work should look at combinations of these factors to improve interpretability of the results. Finally, parameterizing models for emerging pathogen possesses a unique challenge as parameters vary over time and space. Future work should examine how to parameterize complex models where values may not be known or changing over time.

With vaccine supplies usually severely constrained, rapid allocation decisions will need to be made while epidemics are ongoing now and in the future. Due to the global impact and magnitude of some pandemics such as the current SARS-CoV-2 pandemic, further political and economic constraints will likely play a large role in allocation decisions. Mathematical modelling can provide insight into optimal allocation strategies that maximize the benefit from each dose. Conclusions from such models should be balanced with ethical considerations on the fairness of allocation that also minimize disparities in access. We show key principles that should be considered in the design of realistic and implementable allocation strategies.

## Supplementary Material

MMC1

## Data Availability

All code to reproduce the analyses can be found on Github at https://github.com/keyajoshi/Vaccine_Allocation
