# Supplementary material for: Comparative performance of between-population vaccine allocation strategies with applications for emerging pandemics
Source: Vaccine. Author manuscript; Available in PMC 2023 Apr 5. (PMC10075509; doi:10.1016/j.vaccine.2022.12.053)
Supplement: MMC1 [file NIHMS1869722-supplement-MMC1.docx]

Comparative performance of between-population vaccine allocation strategies with applications for emerging pandemics

Supporting Information

Keya Joshi^1,*,a^, Eva Rumpler^1,*,a^, Lee Kennedy-Shaffer^a,b^, Rafia Bosan^a^ and Marc Lipsitch^a^

^a^ Center for Communicable Disease Dynamics, Department of Epidemiology, Harvard TH

Chan School of Public Health, 02115 Boston, Massachusetts

^b^ Department of Mathematics & Statistics, Vassar College, 12604 Poughkeepsie, New York

^1^Contributed equally to this work.

^*^Corresponding authors.

# A Supporting Information

## A.1 Supplementary Figures


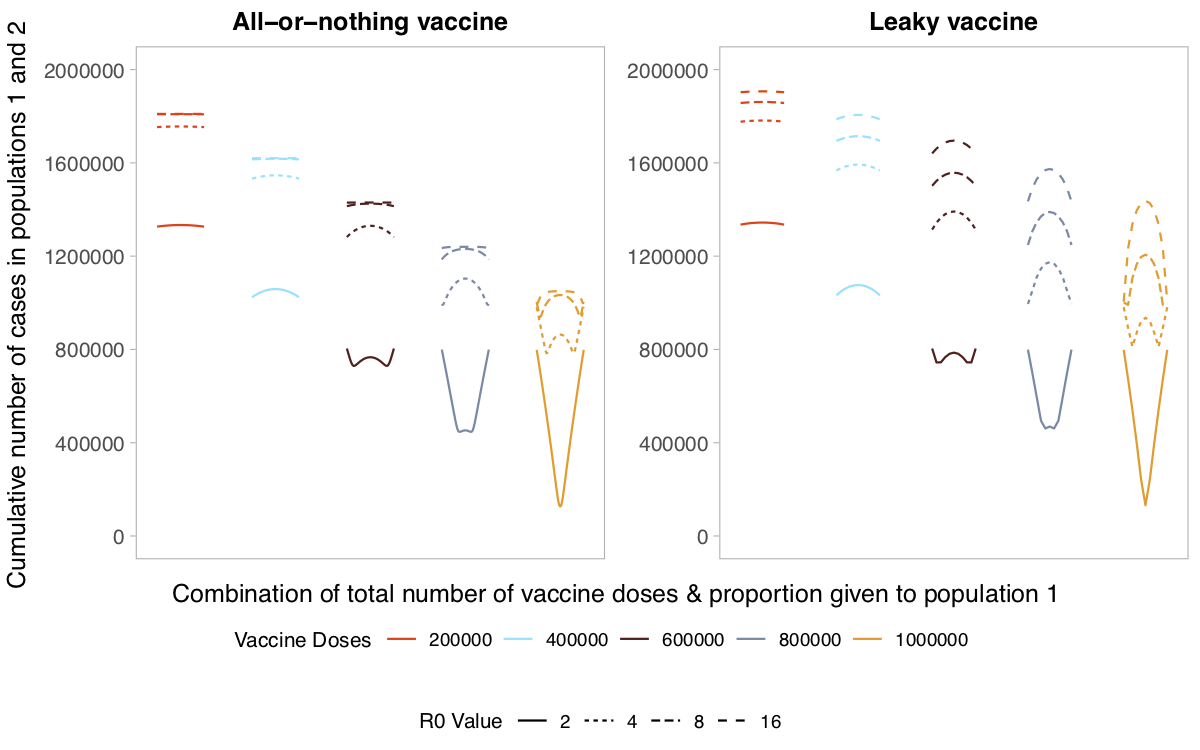


**Figure S1**: Performance of different allocation strategies of a limited vaccine stockpile across two homogeneous populations of equal size (one million individuals) with no underlying immunity and prophylactic vaccination. An all-or-nothing vaccine (left) is compared to a leaky vaccine (right). The panel on the left is equivalent to Figure 1.


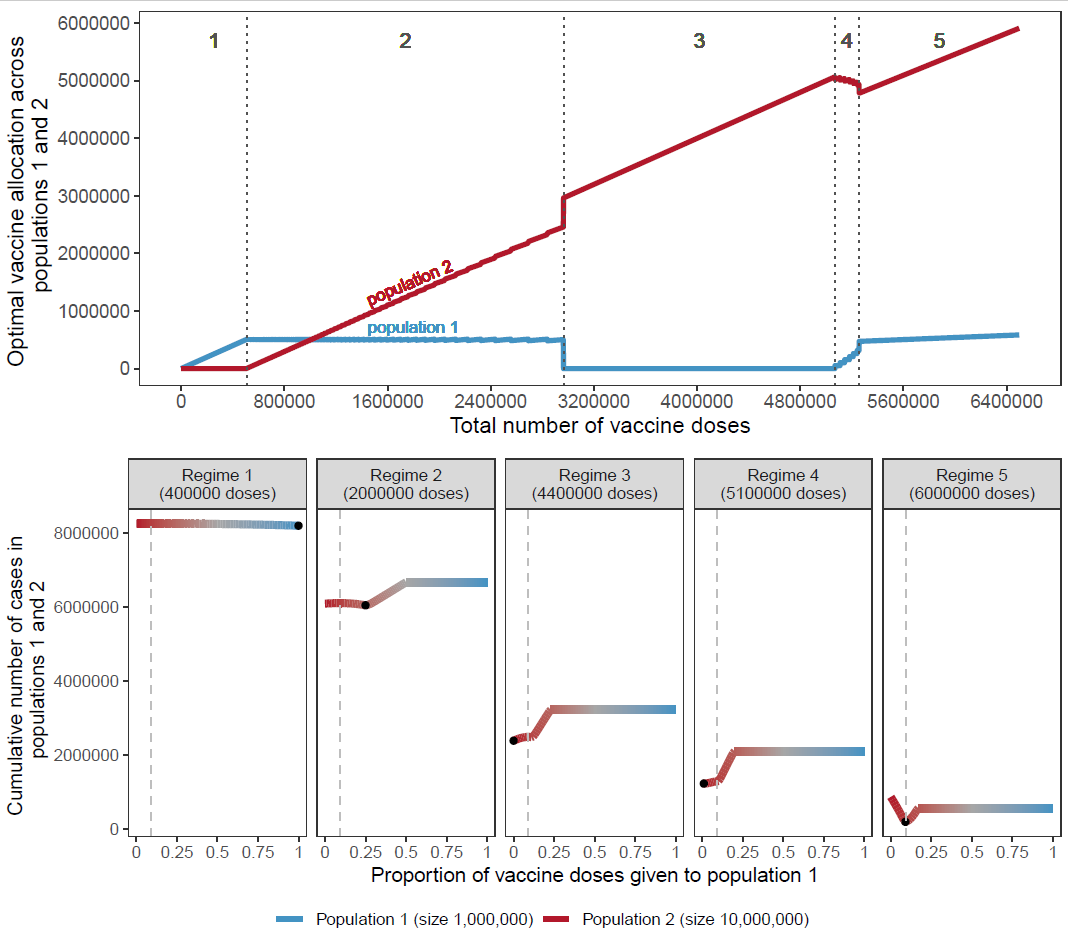


**Figure S2:** **Top**: Optimal allocation strategies of a limited vaccine stockpile across two homogeneous populations of unequal size with no underlying immunity, prophylactic vaccination and an R_0_ of 2. Populations 1 (blue) and 2 (red) have one and ten million individuals, respectively. Dotted vertical lines were added to highlight regimes (1 to 5) showing different vaccine allocation patterns. **Bottom**: Performance of allocation strategies for five different numbers of vaccine doses, representative of the regimes shown in the top half of the Figure. Color coding corresponds to vaccine allocation ranging from giving all doses to population 2 (red) to giving all doses to population 1 (blue). The optimal allocation, the minimal value on each plot, is highlighted by a black point. Dashed vertical lines in the bottom panel represent pro-rata allocation between population 1 and 2.


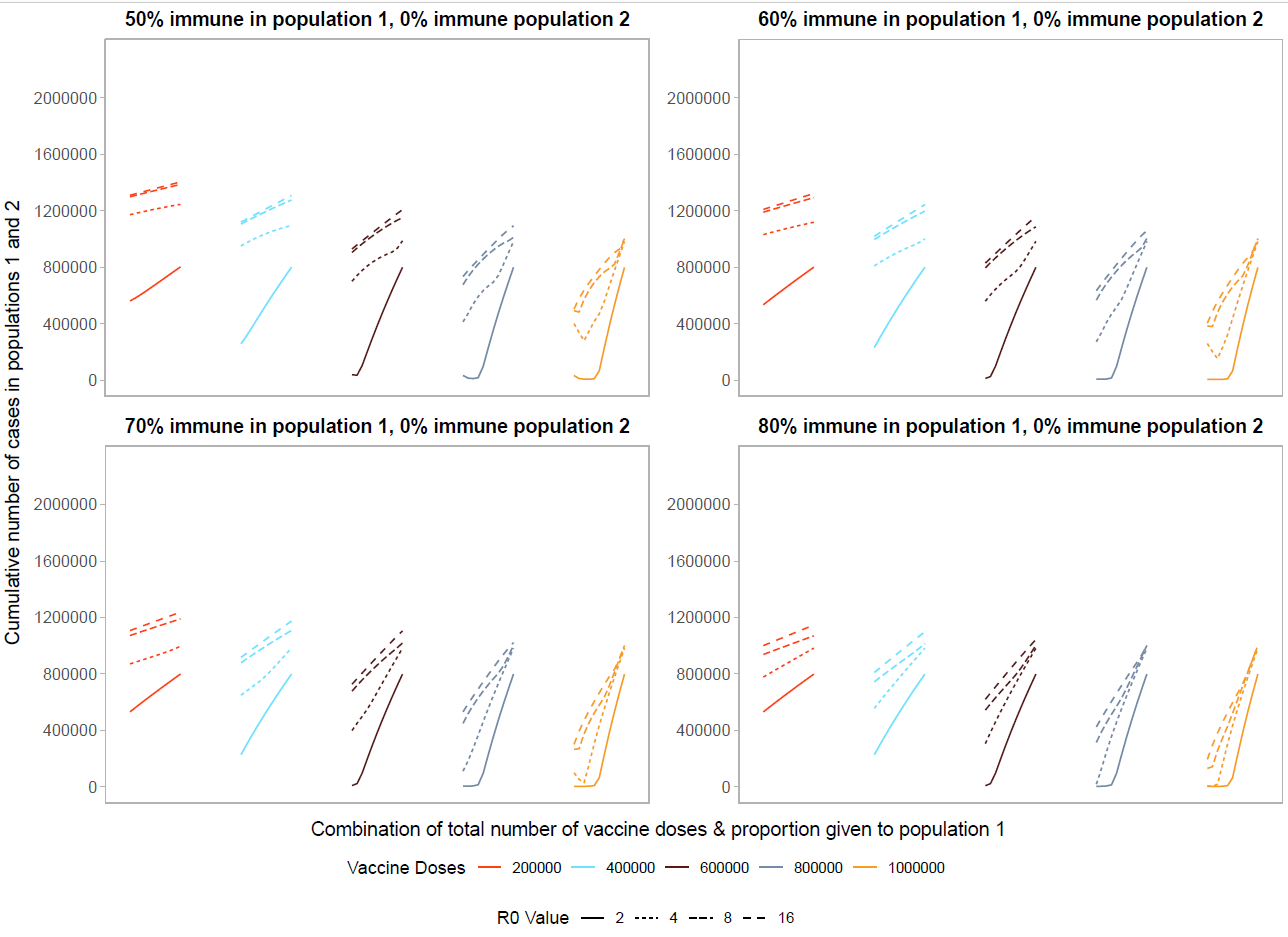


**Figure S3**: Performance of different allocation strategies of a limited vaccine stockpile across two homogeneous populations of equal size (one million individuals) with different underlying immunity, and prophylactic vaccination. We fix population 2 to have no underlying pathogen immunity and vary underlying immunity in population 1 from 50 to 80%. Each color represents a different number of total vaccine doses. Each line represents a different basic reproductive number between 2 and 16.

**
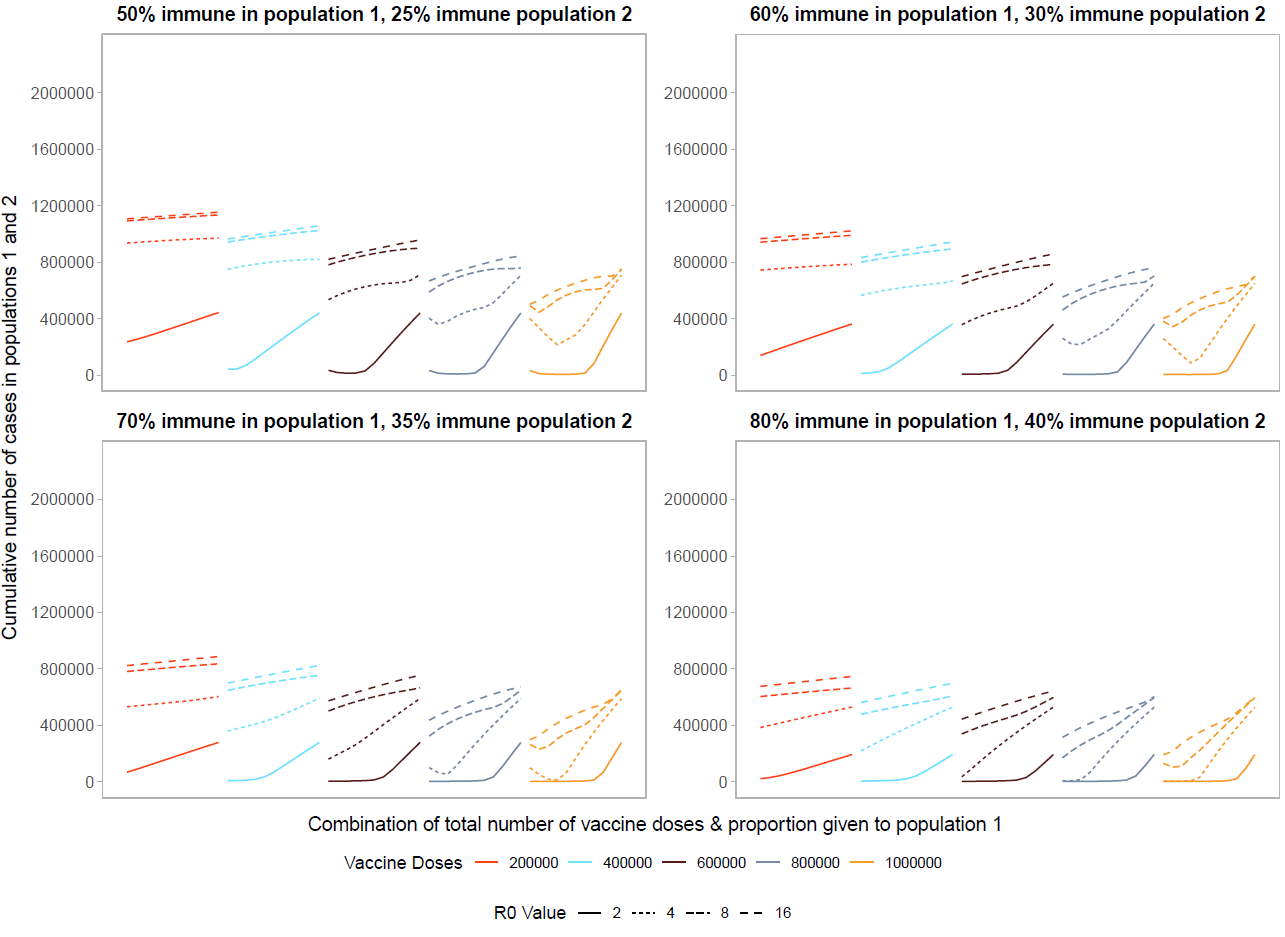
**

**Figure S4** Performance of different allocation strategies of a limited vaccine stockpile across two homogeneous populations of equal size (one million individuals) with different underlying immunity, and prophylactic vaccination. We vary underlying pathogen immunity in population 1 from 50 to 80% and allow population 2 to have half the underlying population immunity of population 1. Each color represents a different number of total vaccine doses. Each line represents a different basic reproductive number between 2 and 16.


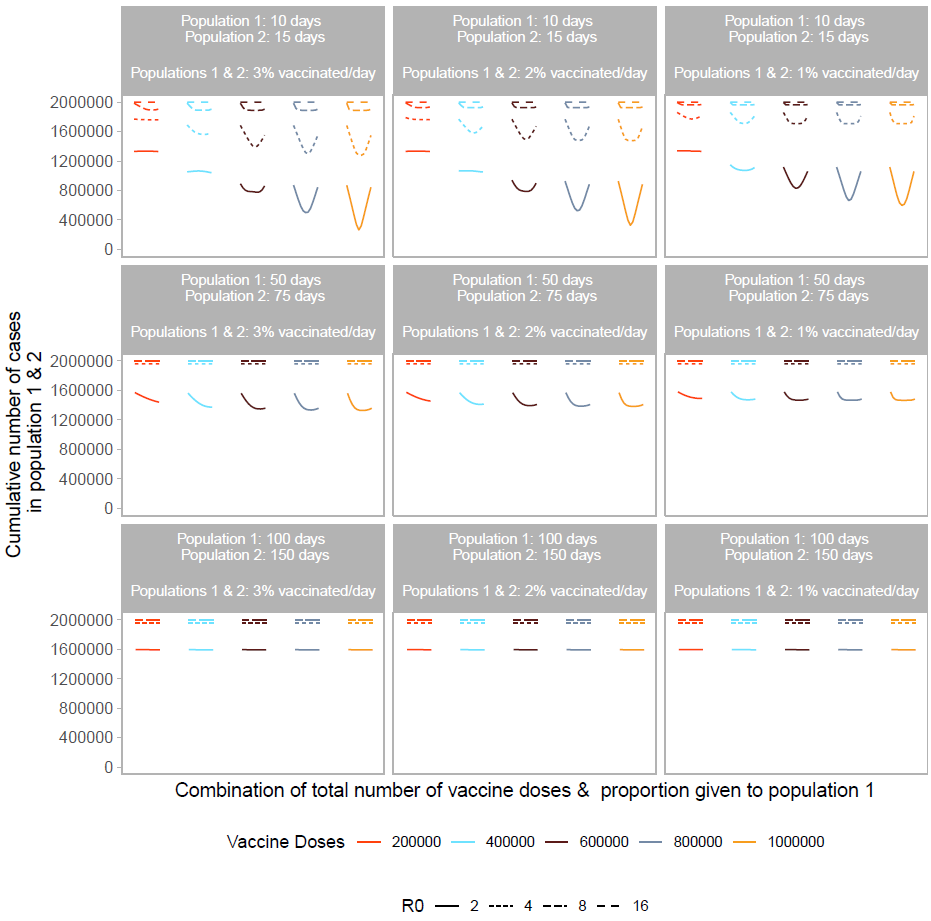


**Figure S5**: Performance of different allocation strategies of a limited vaccine stockpile across two homogeneous populations of equal size (one million individuals) with no underlying immunity, with vaccines rolled out at different speeds and different times after the start of the epidemic. Each color represents a different number of total vaccine doses. Each line represents a different basic reproductive number between 2 and 16. We vary the speed of roll-out with 1, 2, or 3% of the population vaccinated per day across both populations. We allow the timing of vaccine rollout to differ between population 1 and population 2, with rollout starting at 10, 50, or 100 days after the start of the epidemic for population 1 and rollout starting at 15, 75, and 150 days after the start of the epidemic for population 2 (i.e., 1.5 times later than population 1). Each column represents a given roll-out speed while each row represents a different timing.


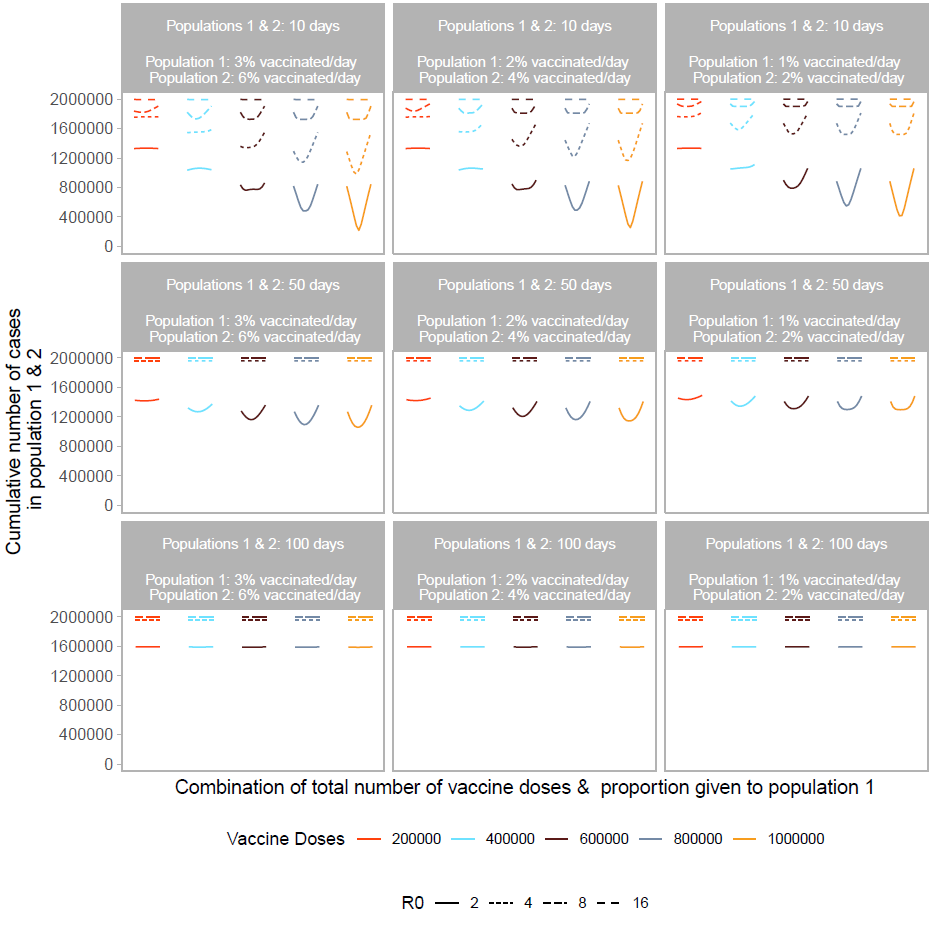


**Figure S6**: Performance of different allocation strategies of a limited vaccine stockpile across two homogeneous populations of equal size (one million individuals) with no underlying immunity, with vaccines rolled out at different speeds and different times after the start of the epidemic. Each color represents a different number of total vaccine doses. Each line represents a different basic reproductive number between 2 and 16. We vary the timing roll-out between 10, 50 or 100 days after the start of the epidemic across both populations. We allow the speed of roll-out to differ between population 1 and population 2, with 1, 2, or 3% of the population vaccinated per day in population 1 and 2, 4, or 6% vaccinated in population 2 (i.e., twice the speed of population 1). Each column represents a given roll-out speed while each row represents a different timing.


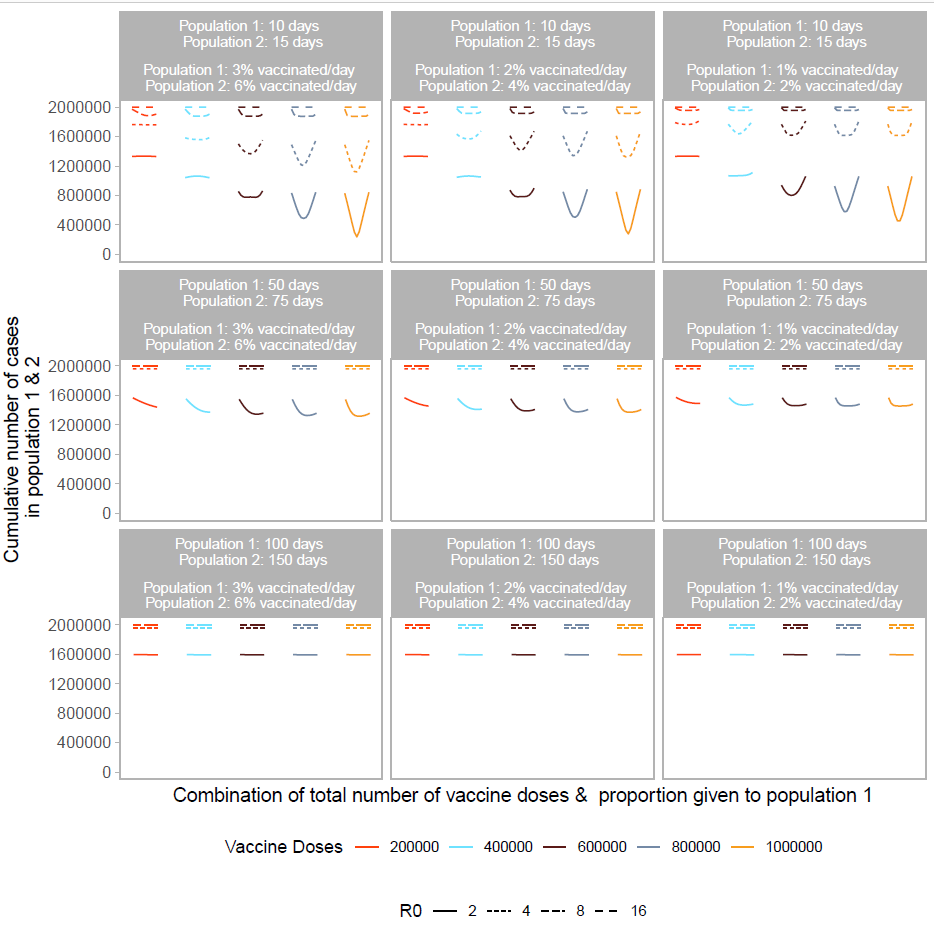


**Figure S7**: Performance of different allocation strategies of a limited vaccine stockpile across two homogeneous populations of equal size (one million individuals) with no underlying immunity, with vaccines rolled out at different speeds and different times after the start of the epidemic. Each color represents a different number of total vaccine doses. Each line represents a different basic reproductive number between 2 and 16. We allow both the timing and speed of rollout to differ between the two populations. We vary timing between 10, 50 or 100 days after the start of the epidemic in population and 15, 75, and 150 days in population 2 (i.e., 1.5 times later than population 1). We additionally vary speed between the two populations with population 1 vaccinating 1, 2, or 3% of the population per day and population 2 vaccinating 2, 4, or 6% of the population per day (i.e., twice the speed of population 1). Each column represents a given roll-out speed while each row represents a different timing.

**Figure S8**: Performance of different allocation strategies of a limited vaccine stockpile across two heterogeneous populations of equal size (one million individuals) with no underlying immunity and prophylactic vaccination. Each color represents a different number of total vaccine doses. Each line represents a different basic reproductive number from 2 to 16. In both the high transmission scenario (top) and high mortality scenario (bottom), 50% of both populations are high risk.

**Figure S9:** Performance of different allocation strategies of a limited vaccine stockpile across two heterogeneous populations of equal size (one million individuals) with no underlying immunity, with vaccines rolled out at different speeds and different times after the start of the epidemic. 25% of both populations are high risk of transmission. We vary the timing of roll-out between 10, 30, 50, or 100 days after the start of the epidemic, and vary the speed of roll-out between 1, 2, or 3% of the population vaccinated per day. Each color represents a different number of total vaccine doses. Each line represents a different basic reproductive number between 2 and 16.


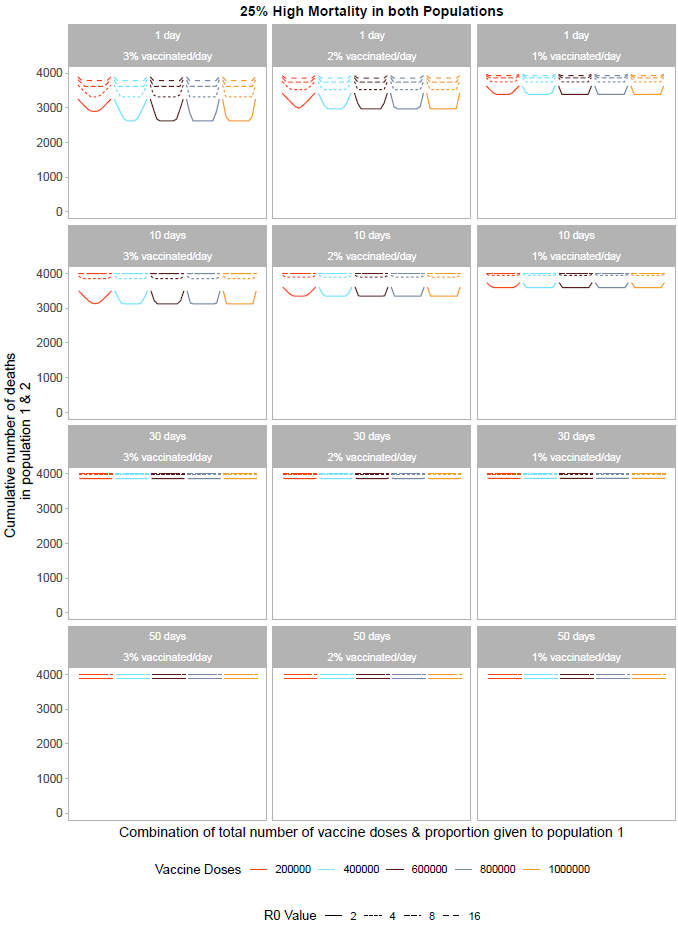


**Figure S10:** Performance of different allocation strategies of a limited vaccine stockpile across two heterogeneous populations of equal size (one million individuals) with no underlying immunity, with vaccines rolled out at different speeds and different times after the start of the epidemic. 25% of both populations are high risk of mortality. We vary the timing of roll-out between 1, 10, 30 or 50 days after the start of the epidemic and vary the speed of roll-out between 1, 2, or 3% of the population vaccinated per day. Each color represents a different number of total vaccine doses. Each line represents a different basic reproductive number between 2 and 16.

**Figure S11:** Performance of different allocation strategies of a limited vaccine stockpile across two heterogeneous populations of equal size (one million individuals) with no underlying immunity, with vaccines rolled out at different speeds and different times after the start of the epidemic. 50% of both populations are high risk of transmission. We vary the timing of roll-out between 10, 30, 50, or 100 days after the start of the epidemic, and vary the speed of roll-out between 1, 2, or 3% of the population vaccinated per day. Each color represents a different number of total vaccine doses. Each line represents a different basic reproductive number between 2 and 16.


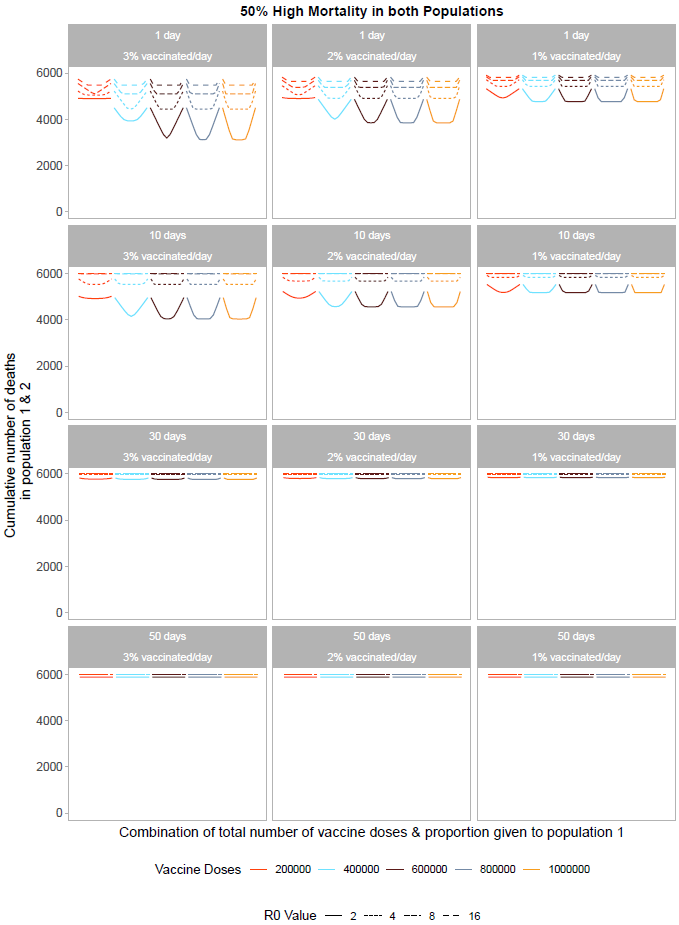


**Figure S12:** Performance of different allocation strategies of a limited vaccine stockpile across two heterogeneous populations of equal size (one million individuals) with no underlying immunity, with vaccines rolled out at different speeds and different times after the start of the epidemic. 50% of both populations are high risk of mortality. We vary the timing of roll-out between 1, 10, 30 or 50 days after the start of the epidemic and vary the speed of roll-out between 1, 2, or 3% of the population vaccinated per day. Each color represents a different number of total vaccine doses. Each line represents a different basic reproductive number between 2 and 16.

**
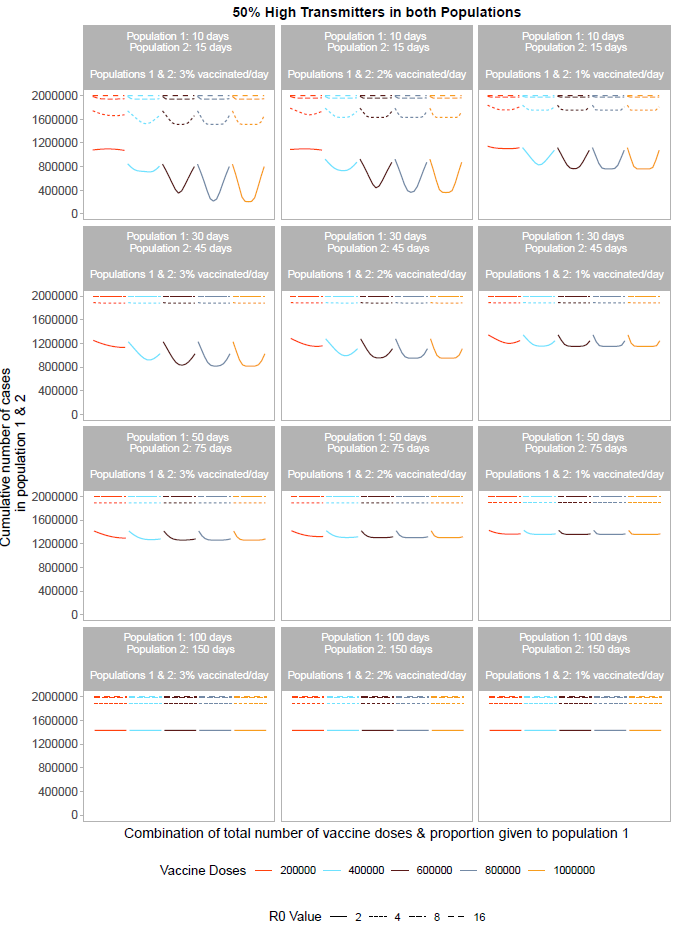
**

**Figure S13:** Performance of different allocation strategies of a limited vaccine stockpile across two heterogeneous populations of equal size (one million individuals) with no underlying immunity, with vaccines rolled out at different speeds and different times after the start of the epidemic. 50% of both populations are high risk of transmission. Each color represents a different number of total vaccine doses. Each line represents a different basic reproductive number between 2 and 16. We vary the speed of roll-out with 1, 2, or 3% of the population vaccinated per day across both populations. We allow the timing of vaccine rollout to differ between population 1 and population 2, with rollout starting at 10, 30, 50, or 100 days after the start of the epidemic for population 1 and rollout starting at 15, 45, 75, and 150 days after the start of the epidemic for population 2 (i.e., 1.5 times later than population 1). Each column represents a given roll-out speed while each row represents a different timing.

**
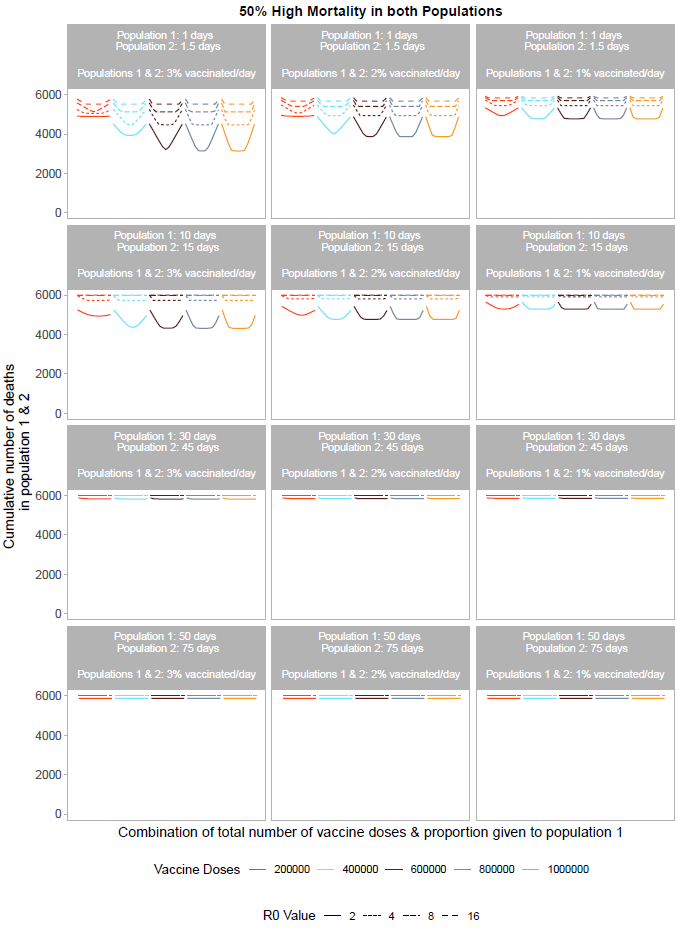
**

**Figure S14:** Performance of different allocation strategies of a limited vaccine stockpile across two heterogeneous populations of equal size (one million individuals) with no underlying immunity, with vaccines rolled out at different speeds and different times after the start of the epidemic. 50% of both populations are high risk of transmission. Each color represents a different number of total vaccine doses. Each line represents a different basic reproductive number between 2 and 16. We vary the speed of roll-out with 1, 2, or 3% of the population vaccinated per day across both populations. We allow the timing of vaccine rollout to differ between population 1 and population 2, with rollout starting at 1, 10, 30, or 50 days after the start of the epidemic for population 1 and rollout starting at 1.5, 15, 45, and 75 days after the start of the epidemic for population 2 (i.e., 1.5 times later than population 1). Each column represents a given roll-out speed while each row represents a different timing.

**
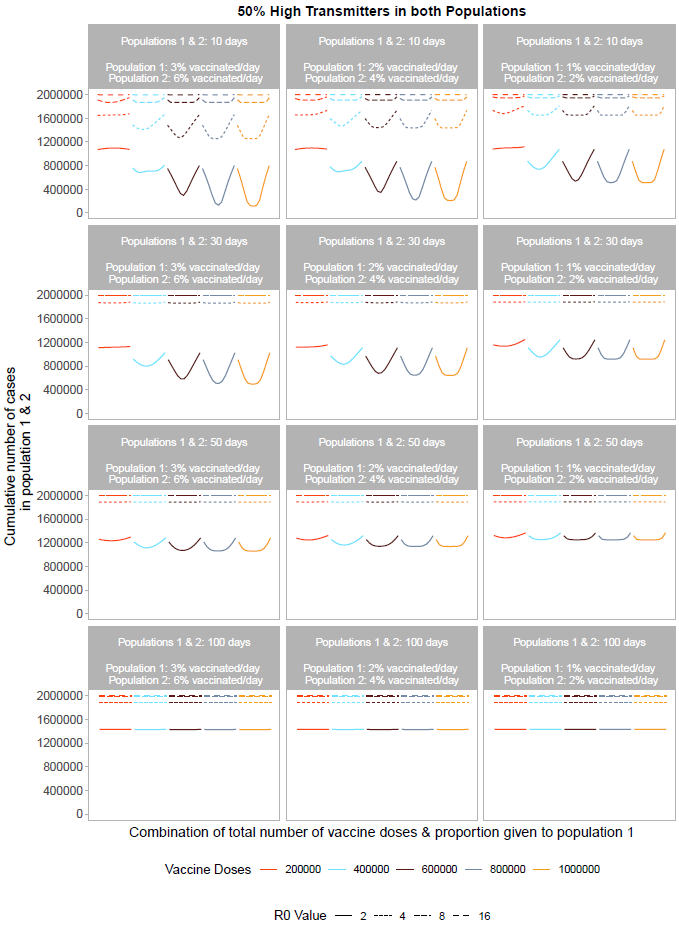
**

**Figure S15**: Performance of different allocation strategies of a limited vaccine stockpile across two heterogeneous populations of equal size (one million individuals) with no underlying immunity, with vaccines rolled out at different speeds and different times after the start of the epidemic. 50% of both populations are high risk of transmission. We vary the timing of roll-out between 10, 30, 50, or 100 days after the start of the epidemic. We allow the speed of roll-out to differ between population 1 and population 2, with 1, 2, or 3% of the population vaccinated per day in population 1 and 2, 4, or 6% vaccinated in population 2 (i.e., twice the speed of population 1). Each column represents a given roll-out speed while each row represents a different timing.

**
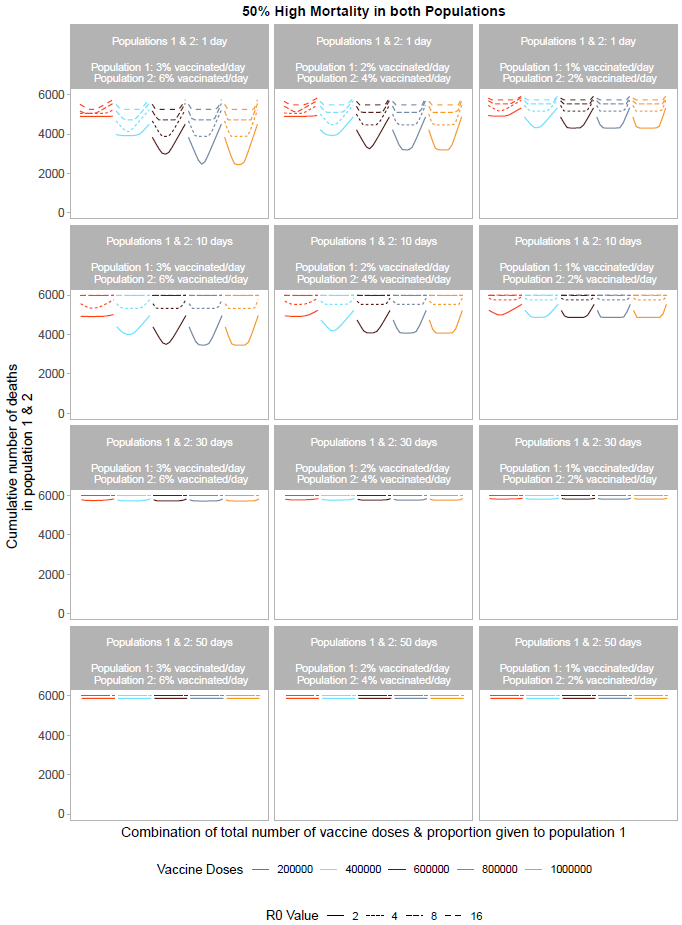
**

**Figure S16**: Performance of different allocation strategies of a limited vaccine stockpile across two heterogeneous populations of equal size (one million individuals) with no underlying immunity, with vaccines rolled out at different speeds and different times after the start of the epidemic. 50% of both populations are high risk of mortality. We vary the timing of roll-out between 1, 10, 30, or 50 days after the start of the epidemic We allow the speed of roll-out to differ between population 1 and population 2, with 1, 2, or 3% of the population vaccinated per day in population 1 and 2, 4, or 6% vaccinated in population 2 (i.e., twice the speed of population 1). Each column represents a given roll-out speed while each row represents a different timing.

**Figure S17:** Performance of different allocation strategies of a limited vaccine stockpile across two homogeneous populations of equal size (one million individuals) with no underlying immunity, prophylactic vaccination, and an R_0_ = 2. Each color represents a different number of total vaccine doses. Each line represents a different vaccine efficacy value from 50 to 90%.

**Figure S18:** Performance of different allocation strategies of a limited vaccine stockpile across two homogeneous populations of equal size (one million individuals) with different underlying immunity, prophylactic vaccination, and an R_0_ = 2. We vary underlying immunity from 0 to 40%. The proportion if immune individuals is identical in both populations. Each color represents a different number of total vaccine doses. Each line represents a different vaccine efficacy value from 50 to 90%. The panel on the top left is equivalent to Figure S17.


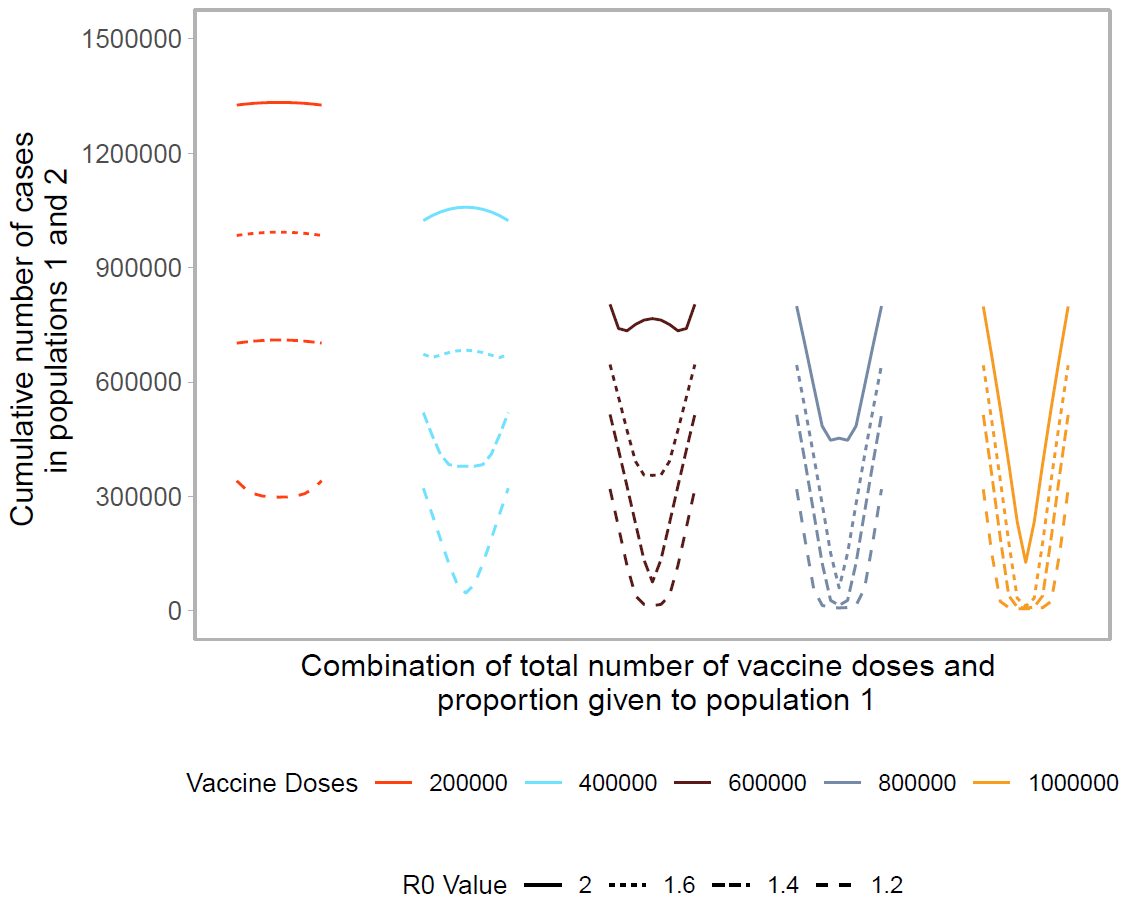


**Figure S19**: Performance of different allocation strategies of a limited vaccine stockpile across two homogeneous population of equal size with no underlying immunity and prophylactic vaccination. Both populations have one million individuals. Each color represents a different number of total vaccine doses. Each line represents a different basic reproductive number from 1.2 to 2. The solid line across for each number of vaccine doses is equivalent to that seen in Figure 1.


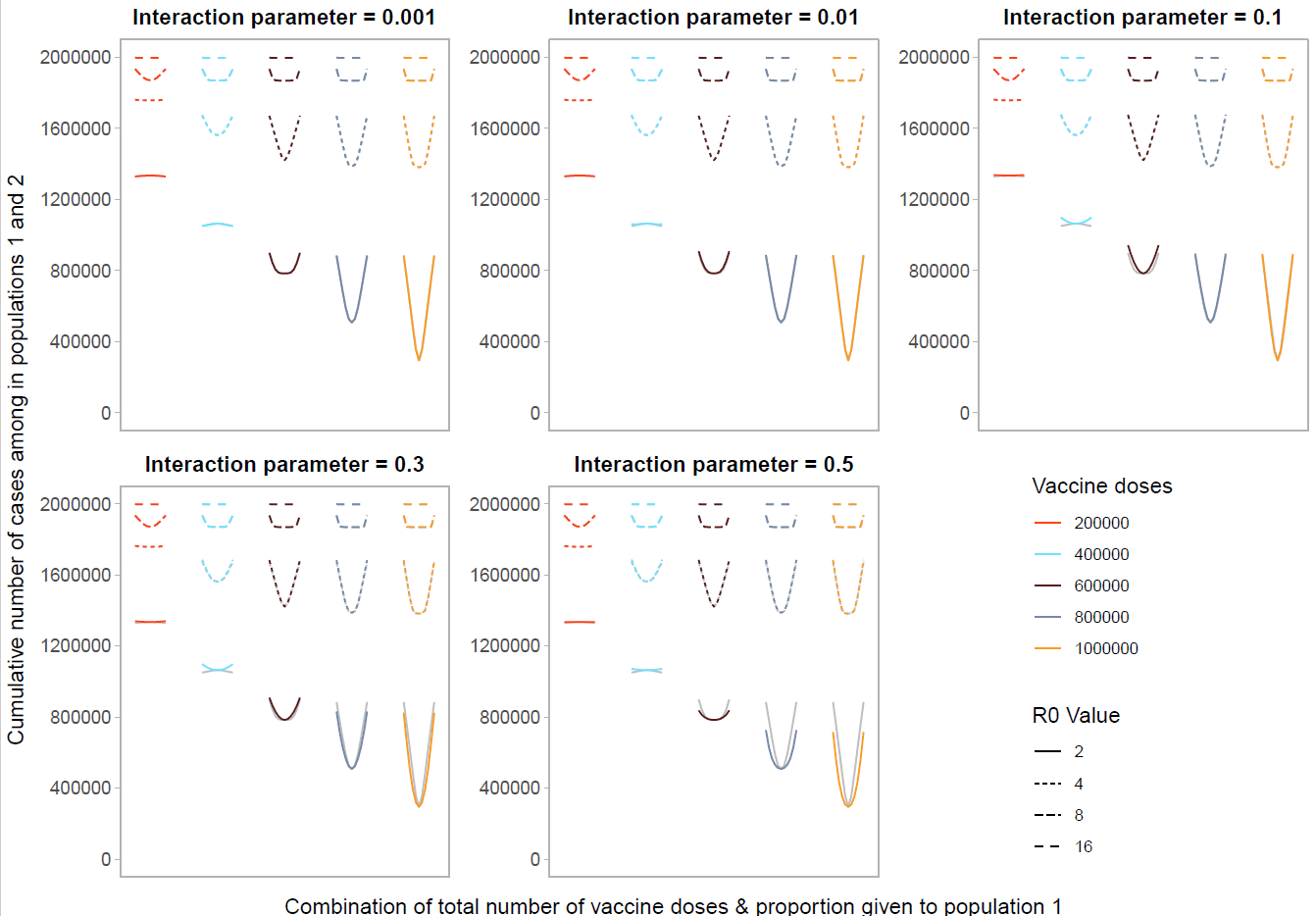


**Figure S20**: Performance of different allocation strategies of a limited vaccine stockpile across two homogeneous populations of equal size (one million individuals) with no underlying immunity, with vaccines rolled out starting 10 days after the start of the epidemic with 2% of the population vaccinated each day. The populations are allowed to interact of varying degree (i=0 corresponds no interaction and i=0.5 corresponds to complete interaction or perfect mixing between the two populations). The grey lines in each panel are equivalent to Figure 1 and represent a scenario without interaction between the two populations.

| **Figure** | **Population risk** | **Population size** | **Underlying immunity** | **Vaccination**  **strategy** | **Vaccine**  **efficacy** | **Vaccine type** | **R_0_**  **values** | **Interaction** |
| --- | --- | --- | --- | --- | --- | --- | --- | --- |
| 1 | Homogeneous | Equal | No | Prophylactic | 95% | All-or-nothing | 2, 4, 8, 16 | No |
| 2 | Homogeneous | Unequal (2x) | No | Prophylactic | 95% | All-or-nothing | 2 | No |
| 3 | Homogeneous | Equal | 0-40% | Prophylactic | 95% | All-or-nothing | 2, 4, 8, 16 | No |
| 4 | Homogeneous | Equal | No | Rollout (same speed/  timing across populations) | 95% | All-or-nothing | 2, 4, 8, 16 | No |
| 5 | Heterogeneous (25% high risk) | Equal | No | Prophylactic | 95% | All-or-nothing | 2, 4, 8, 16 | No |
| S1 | Homogeneous | Equal | No | Prophylactic | 95% | Leaky | 2,4,8,16 | No |
| S2 | Homogeneous | Unequal (10x) | No | Prophylactic | 95% | All-or-nothing | 2 | No |
| S3 | Homogeneous | Equal | 50-80% | Prophylactic | 95% | All-or-nothing | 2,4,8,16 | No |
| S4 | Homogeneous | Equal | 50-80% population 1,  25-40% population 2 | Prophylactic | 95% | All-or-nothing | 2,4,8,16 | No |
| S5 | Homogeneous | Equal | No | Rollout (same speed/population 2 timing 1.5x later) | 95% | All-or-nothing | 2,4,8,16 | No |
| S6 | Homogeneous | Equal | No | Rollout (same timing/population 2 speed 2x) | 95% | All-or-nothing | 2,4,8,16 | No |
| S7 | Homogeneous | Equal | No | Rollout (different speed/timing across populations) | 95% | All-or-nothing | 2,4,8,16 | No |
| S8 | Heterogeneous (50% high risk) | Equal | No | Prophylactic | 95% | All-or-nothing | 2,4,8,16 | No |
| S9 | Heterogeneous (25% high risk transmission) | Equal | No | Rollout (same speed/  timing across populations | 95% | All-or-nothing | 2,4,8,16 | No |
| S10 | Heterogeneous (25% high risk mortality) | Equal | No | Rollout (same speed/  timing across populations | 95% | All-or-nothing | 2,4,8,16 | No |
| S11 | Heterogeneous (50% high risk transmission) | Equal | No | Rollout (same speed/  timing across populations | 95% | All-or-nothing | 2,4,8,16 | No |
| S12 | Heterogeneous (50% high risk mortality) | Equal | No | Rollout (same speed/  timing across populations | 95% | All-or-nothing | 2,4,8,16 | No |
| S13 | Heterogeneous (50% high risk transmission) | Equal | No | Rollout (same speed/population 2 timing 1.5x later) | 95% | All-or-nothing | 2,4,8,16 | No |
| S14 | Heterogeneous (50% high risk mortality | Equal | No | Rollout (same speed/population 2 timing 1.5x later) | 95% | All-or-nothing | 2,4,8,16 | No |
| S15 | Heterogeneous (50% high risk transmission) | Equal | No | Rollout (same timing/population 2 speed 2x) | 95% | All-or-nothing | 2,4,8,16 | No |
| S16 | Heterogeneous (50% high risk mortality | Equal | No | Rollout (same timing/population 2 speed 2x) | 95% | All-or-nothing | 2,4,8,16 | No |
| S17 | Homogeneous | Equal | No | Prophylactic | 50-90% | All-or-nothing | 2 | No |
| S18 | Homogeneous | Equal | 0-40% | Prophylactic | 50-90% | All-or-nothing | 2 | No |
| S19 | Homogeneous | Equal | No | Prophylactic | 95% | All-or-nothing | 1.2, 1.4, 1.6, 1.8, 2 | No |
| S20 | Homogeneous | Equal | No | Rollout (same speed/  timing across populations) | 95% | All-or-nothing | 2,4,8,16 | Yes |

**Table S1:** Description of the variables considered for each figure.

## A.2 Model and Parameters

**A.2.1 SEIR model equations for two non-interacting populations**

**Population 1**

$$\frac{dS_{1}}{dt}=-\beta S_{1}I_{1}$$

$$\frac{dE_{1}}{dt}=\beta S_{1}I_{1}-\sigma E_{1}$$

$$\frac{dI_{1}}{dt}=\sigma E_{1}-\nu I_{1}$$

$$\frac{dR_{1}}{dt}=\nu I_{1}$$

**Population 2**

$$\frac{dS_{2}}{dt}=-\beta S_{2}I_{2}$$

$$\frac{dE_{2}}{dt}=\beta S_{2}I_{2}-\sigma E_{2}$$

$$\frac{dI_{2}}{dt}=\sigma E_{2}-\nu I_{2}$$

$$\frac{dR_{2}}{dt}=\nu I_{2}$$

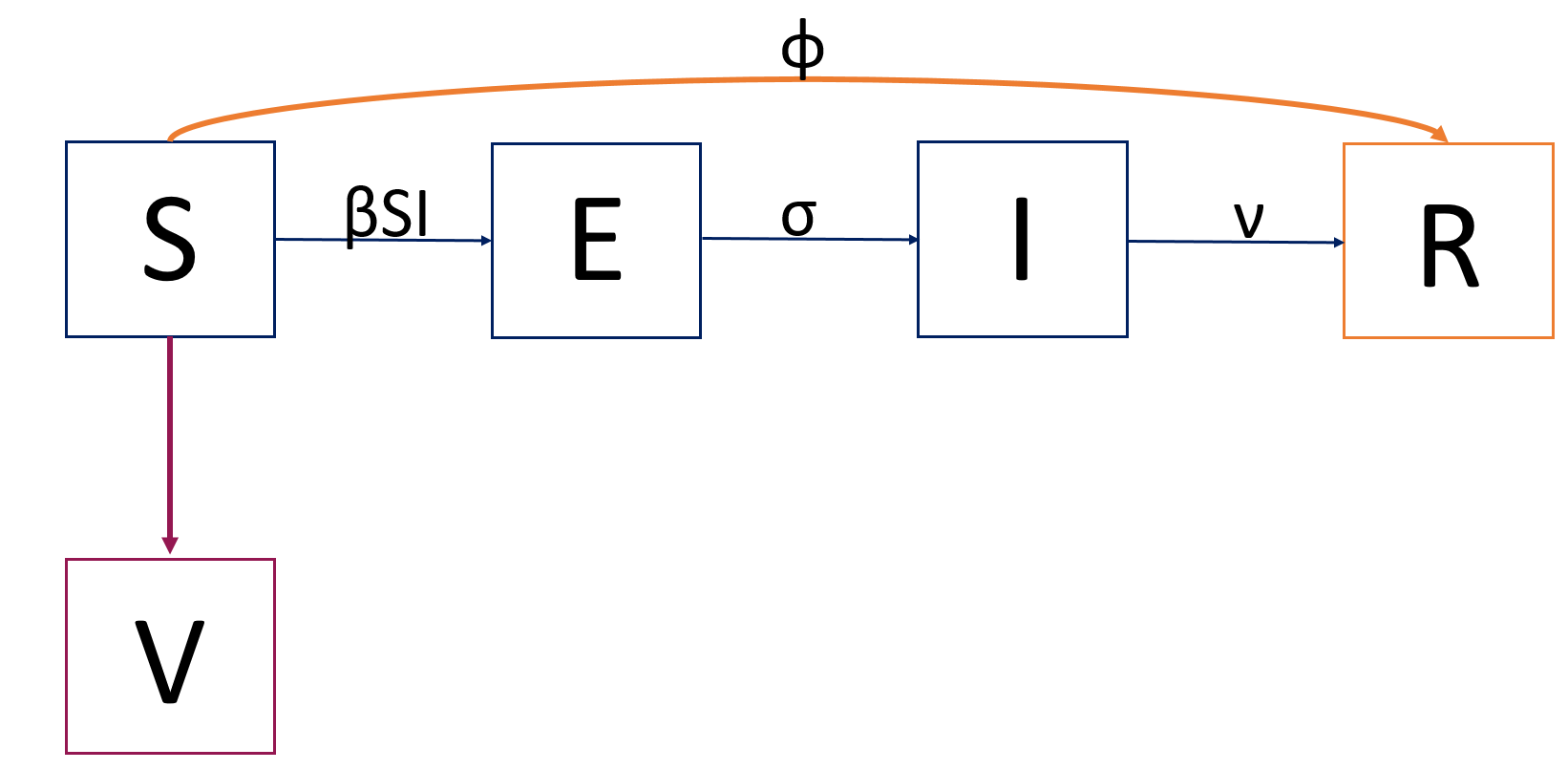


**Figure S21**: SEIR model incorporating underlying immunity (in orange) and continuous roll-out of vaccination (in purple).

**A.2.2 Parameters**

| **Parameter** | **Definition** | **Value**  **[Sensitivity Analysis]** | **Citation** |
| --- | --- | --- | --- |
| *σ*^−1^ | Latent period | 3 days | [1,2] |
| *ν*^−1^ | Infectious period | 5 days | [1,2] |
| *R*_0_ | Basic reproduction number | 2, 4, 8, 16  [1.2, 1.4, 1.6, 1.8, 2] | [3–5] |
| *τ* | Vaccine efficacy | 0.95  [0.5-0.9] | [6,7] |
| *v* | Number of vaccine doses available | 1,000,000 |  |
| *pv*_1_ | Proportion of the total vaccines given to population 1 | [0,1] |  |
| *φ_1_* | Proportion of the population immune in population 1 | 0, 0.1, 0.2, 0.4,  [0.5, 0.6, 0.7, 0.8] | [8,9] |
| *φ_2_* | Proportion of the population immune in population 2 | 0  [0.25, 0.3, 0.35, 0.4] |  |
| Rollout time | Vaccine roll-out time | 1, 10, 30, 50, 100 days |  |
| Rollout speed | Vaccine roll-out speed | 1-3% per day  [2,4,6] |  |
| *ph_i_* | Proportion of high-risk individuals in population *i* | 0.25, 0.5 |  |
| *i* | Proportion of interaction in population 1 and 2 | [0,0.5] |  |
| *s_h_* | Proportion of high-risk infected individuals that do not die in the model accounting for heterogeneous risk of mortality | 0.995 | [10] |
| *s_l_* | Proportion of low-risk infected individuals that do not die in the model accounting for heterogeneous risk of mortality | 0.999 | [10] |

**A.2.3 SEIR model equations for two interacting populations**

We extend the equations from the original SEIR model to allow for interaction between population 1 and population 2.

**Population 1**

$$\frac{dS_{1}}{dt}=-\beta S_{1}\left[ \left( 1-i \right)I_{1}+iI_{2} \right]$$

$$\frac{dE_{1}}{dt}=\beta S_{1}\left[ \left( 1-i \right)I_{1}+iI_{2} \right]-\sigma E_{1}$$

$$\frac{dI_{1}}{dt}=\sigma E_{1}-\nu I_{1}$$

$$\frac{dR_{1}}{dt}=\nu I_{1}$$

**Population 2**

$$\frac{dS_{2}}{dt}=-\beta S_{2}\left[ \left( 1-i \right)I_{2}+iI_{1} \right]$$

$$\frac{dE_{2}}{dt}=\beta S_{2}\left[ \left( 1-i \right)I_{2}+iI_{1} \right]-\sigma E_{2}$$

$$\frac{dI_{2}}{dt}=\sigma E_{2}-\nu I_{2}$$

$$\frac{dR_{2}}{dt}=\nu I_{2}$$

**A.2.4 SEIR model equations for high-risk of transmission**

We extend the equations from the original SEIR model to allow individuals at higher risk of transmission.

**Population 1**

$$\frac{dS_{1H}}{dt}=-\beta_{HH}S_{1H}I_{1H}-\beta_{HL}S_{1H}I_{1L}$$

$$\frac{dE_{1H}}{dt}=\beta_{HH}S_{1H}I_{1H}+\beta_{HL}S_{1H}I_{1L}-\sigma E_{1H}$$

$$\frac{dI_{1H}}{dt}=\sigma E_{1H}-\nu I_{1H}$$

$$\frac{dR_{1H}}{dt}=s_{h}\nu I_{1H}$$

$$\frac{dS_{1L}}{dt}=-\beta_{LL}S_{1L}I_{1L}-\beta_{LH}S_{1L}I_{1H}$$

$$\frac{dE_{1L}}{dt}=\beta_{LL}S_{1L}I_{1L}+\beta_{LH}S_{1L}I_{1H}-\sigma E_{1L}$$

$$\frac{dI_{1L}}{dt}=\sigma E_{1L}-\nu I_{1L}$$

$$\frac{dR_{1L}}{dt}=s_{l}\nu I_{1L}$$

**Population 2**

$$\frac{dS_{2H}}{dt}=-\beta_{HH}S_{2H}I_{2H}-\beta_{HL}S_{2H}I_{2L}$$

$$\frac{dE_{2H}}{dt}=\beta_{HH}S_{2H}I_{2H}+\beta_{HL}S_{2H}I_{2L}-\sigma E_{2H}$$

$$\frac{dI_{2H}}{dt}=\sigma E_{2H}-\nu I_{2H}$$

$$\frac{dR_{2H}}{dt}=s_{h}\nu I_{2H}$$

$$\frac{dS_{2L}}{dt}=-\beta_{LL}S_{2L}I_{2L}-\beta_{LH}S_{2L}I_{2H}$$

$$\frac{dE_{2L}}{dt}=\beta_{LL}S_{2L}I_{2L}+\beta_{LH}S_{2L}I_{2H}-\sigma E_{2L}$$

$$\frac{dI_{2L}}{dt}=\sigma E_{2L}-\nu I_{2L}$$

$$\frac{dR_{2L}}{dt}=s_{l}\nu I_{2L}$$

**A.2.5 SEIR model equations for high-risk of mortality**

We extend the equations from the original SEIR model to allow individuals at higher risk of death.

**Population 1**

$$\frac{dS_{1H}}{dt}=-\beta S_{1H}I_{1H}-\beta S_{1H}I_{1L}$$

$$\frac{dE_{1H}}{dt}=\beta S_{1H}I_{1H}+\beta S_{1H}I_{1L}-\sigma E_{1H}$$

$$\frac{dI_{1H}}{dt}=\sigma E_{1H}-\nu I_{1H}$$

$$\frac{dR_{1H}}{dt}=s_{h}\nu I_{1H}$$

$$\frac{dD_{1H}}{dt}=\left( 1-s_{h} \right)\nu I_{1H}$$

$$\frac{dS_{1L}}{dt}=-\beta S_{1L}I_{1L}-\beta S_{1L}I_{1H}$$

$$\frac{dE_{1L}}{dt}=\beta S_{1L}I_{1L}+\beta S_{1L}I_{1H}-\sigma E_{1L}$$

$$\frac{dI_{1L}}{dt}=\sigma E_{1L}-\nu I_{1L}$$

$$\frac{dR_{1L}}{dt}=s_{l}\nu I_{1L}$$

$$\frac{dD_{1L}}{dt}=\left( 1-s_{l} \right)\nu I_{1L}$$

**Population 2**

$$\frac{dS_{2H}}{dt}=-\beta S_{2H}I_{2H}-\beta S_{2H}I_{2L}$$

$$\frac{dE_{2H}}{dt}=\beta S_{2H}I_{2H}+\beta S_{2H}I_{2L}-\sigma E_{2H}$$

$$\frac{dI_{2H}}{dt}=\sigma E_{2H}-\nu I_{2H}$$

$$\frac{dR_{2H}}{dt}=s_{h}\nu I_{2H}$$

$$\frac{dD_{2H}}{dt}=\left( 1-s_{h} \right)\nu I_{2H}$$

$$\frac{dS_{2L}}{dt}=-\beta S_{2L}I_{2L}-\beta S_{2L}I_{2H}$$

$$\frac{dE_{2L}}{dt}=\beta S_{2L}I_{2L}+\beta S_{2L}I_{2H}-\sigma E_{2L}$$

$$\frac{dI_{2L}}{dt}=\sigma E_{2L}-\nu I_{2L}$$

$$\frac{dR_{2L}}{dt}=s_{l}\nu I_{2L}$$

$$\frac{dD_{2L}}{dt}=\left( 1-s_{l} \right)\nu I_{2L}$$

**A.2.6 Global *R*_0_ calculation for heterogeneous transmission**

To identify the global basic reproduction number for the population in simulations with multiple types of individuals in the population, we use the next generation matrix for the SEIR model [11], with two compartments for each of the SEIR components, one for the high-transmitters individuals and one for low-transmitter individuals. Letting
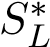
 and
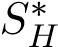
 denote the disease-free-equilibrium proportion of individuals in the low-transmitters and high-transmitters susceptible compartments, respectively, and letting *β_LL_* be the force of transmission from one low-transmitter individual to another, *β_LH_* from a high-transmitter individual to a low-transmitter individual, *β_HL_* from a low-transmitter individual to a high-transmitter individual, and *β_HH_* from one high-transmitter individual to another, we get the components of the next generation matrix:

$$F=\left( \begin{matrix} \begin{matrix} 0 & 0 \\ 0 & 0 \end{matrix} & \begin{matrix} \beta_{LL}S_{L}^{*} & \beta_{LH}S_{L}^{*} \\ \beta_{HL}S_{H}^{*} & \beta_{HH}S_{H}^{*} \end{matrix} \\ \begin{matrix} 0 & 0 \\ 0 & 0 \end{matrix} & \begin{matrix} 0 & 0 \\ 0 & 0 \end{matrix} \end{matrix} \right)$$

$$V=\left( \begin{matrix} \sigma& 0 & 0 & 0 \\ 0 & \sigma& 0 & 0 \\ -\sigma& 0 & \nu& 0 \\ 0 & -\sigma& 0 & \nu\end{matrix} \right)$$

And thus:

$$V^{-1}=\left( \begin{matrix} \sigma^{-1} & 0 & 0 & 0 \\ 0 & \sigma^{-1} & 0 & 0 \\ \nu^{-1} & 0 & \nu^{-1} & 0 \\ 0 & \nu^{-1} & 0 & \nu^{-1} \end{matrix} \right)$$

$${FV}^{-1}=\left( \begin{matrix} \nu^{-1}\beta_{LL}S_{L}^{*} & \nu^{-1}\beta_{LH}S_{L}^{*} & \nu^{-1}\beta_{LL}S_{L}^{*} & \nu^{-1}\beta_{LH}S_{L}^{*} \\ \nu^{-1}\beta_{HL}S_{H}^{*} & \nu^{-1}\beta_{HH}S_{H}^{*} & \nu^{-1}\beta_{HL}S_{H}^{*} & \nu^{-1}\beta_{HH}S_{H}^{*} \\ 0 & 0 & 0 & 0 \\ 0 & 0 & 0 & 0 \end{matrix} \right)$$

The spectral radius of *FV* ^−1^ is then given by the spectral radius of the upper left 2×2 submatrix:

$$\rho\left( {FV}^{-1} \right)=max\left\{ \left| \lambda\right|:\left| \begin{matrix} \nu^{-1}\beta_{LL}S_{L}^{*}-\lambda& \nu^{-1}\beta_{LH}S_{L}^{*} \\ \nu^{-1}\beta_{HL}S_{H}^{*} & \nu^{-1}\beta_{HH}S_{H}^{*}-\lambda\end{matrix} \right|=0 \right\}$$

$$=max\left\{ \left| \lambda\right|:\left( \beta_{LL}S_{L}^{*}-\lambda\nu\right)\left( \beta_{HH}S_{H}^{*}-\lambda\nu\right) -\beta_{LH}S_{L}^{*}\beta_{HL}S_{H}^{*}=0 \right\}$$

$$=max\left\{ \left| \lambda\right|:\left( \lambda\nu\right)^{2}-\left( \beta_{LL}S_{L}^{*}+\beta_{HH}S_{H}^{*} \right)\left( \lambda\nu\right)+\beta_{LL}S_{L}^{*}\beta_{HH}S_{H}^{*}- \beta_{LH}S_{L}^{*}\beta_{HL}S_{H}^{*}=0 \right\}$$

$$=max\left\{ \left| \lambda\right|:\lambda\nu=\frac{\beta_{LL}S_{L}^{*}+\beta_{HH}S_{H}^{*}}{2}\pm\frac{\sqrt{\left( \beta_{LL}S_{L}^{*}+\beta_{HH}S_{H}^{*} \right)^{2}-4\left( \beta_{LL}S_{L}^{*}+\beta_{HH}S_{H}^{*}-\beta_{LH}S_{L}^{*}\beta_{HL}S_{H}^{*} \right)}}{2} \right\}$$

$$=\frac{\beta_{LL}S_{L}^{*}+\beta_{HH}S_{H}^{*}+\sqrt{\left( -\beta_{LL}S_{L}^{*}-\beta_{HH}S_{H}^{*} \right)^{2}+4\beta_{LH}S_{L}^{*}\beta_{HL}S_{H}^{*}}}{2\nu}$$

$$=\frac{R_{LL}+R_{HH}+\sqrt{\left( R_{LL}-R_{HH} \right)^{2}+4R_{LH}R_{HL}}}{2}$$

This value is the global *R*_0_.

*R_HL_* and *R_LH_* represent the number of secondary infections in high-transmitters generated by an infected low-transmitter and the number of secondary infections in a low-transmitter generated by an infected high-transmitter, respectively. *R_HH_* and *R_LL_* represent the number of secondary infections in high-transmitter members generated by an infected high-transmitter and the number of secondary infections in a low-transmitter generated by an infected low-transmitter.

**A.2.7 Vaccine Allocation decision rules**

Below we describe the decision rules when allocating a limited number of vaccines to two populations with heterogeneous risk structure.

1. Assign doses to either population 1 or population 2. This is determined by the *pv* parameter.
2. If the number of vaccine doses is greater than the population size (*v* × *pv > N*_1_) everyone in population 1 is vaccinated, and the leftover doses are assigned to the second population.
3. Once doses are assigned to each population, within each population we first assign all doses to the high-risk individuals and give any doses left to the low-risk individuals. This is accomplished by checking whether the number of doses are sufficient to cover all of the high-risk individuals in that population (i.e., *N*_1_ × *ph*_1_ *< v* × *pv*_1_). If not, we assign all of the doses to high-risk individuals and none to low-risk individuals. If the number of doses are sufficient to cover all high-risk individuals, all of those individuals are vaccinated and the remaining doses are assigned to low-risk individuals.

## A.3 Literature

**A.3.1 Optimal allocation across populations papers**

https://docs.google.com/spreadsheets/d/1NsWWBcztpGG4IpU2U65cUUMryNW9IoKQnoWyd6zig50/edit?usp=sharing

**A.3.2 Details on optimal allocation threshold**

Duijzer et al. [12] identify many features of the direct and indirect effect of vaccination that determine the optimal threshold to which to vaccinate populations. The authors seek to minimize the final size of the epidemic or, equivalently, maximize the total number of people who escape infection. When the number of vaccine doses available is less than the herd immunity threshold, this is also equivalent to maximizing the number of susceptible individuals remaining at the end of the epidemic, i.e., the number of unvaccinated individuals who escape infection, denoted the “herd effect.” They determine that the herd effect, as a function of the vaccination fraction *f* within a population, denoted *G*(*f*), has a predictable structure: it is increasing and convex for a low value of *f*, until *f*^¯^. From *f*^¯^ to *f*^∗^, it is increasing and concave. Above *f*^∗^, *G* is a decreasing function. *f*^∗^ is equivalent to the herd immunity threshold, equal to 1 − 1*/R*_0_ in a fully susceptible population. Vaccinating beyond *f*^∗^ decreases the herd effect, as individuals who would be (somewhat) protected through herd immunity are instead vaccinated and protected directly instead. This convex-concave structure occurs because, for low values of *f*, the epidemic peak is delayed in addition to being smaller in magnitude. Whereas for values of *f* closer to *f*^∗^, the epidemic peak is advanced and smaller in magnitude. As *f* increases to *f*^∗^, this earlier peak continues to advance, leading to a decline in the increase in the herd effect and a concave *G* function. The authors identify another quantity: the dose-optimal vaccination fraction, *f*^˜^, where *f*^¯^≤ *f*^˜^≤ *f*^∗^. The dose-optimal vaccination fraction maximizes the increase in herd effect per dose of vaccine. They find that for multiple non-interacting populations, the optimal allocation is to vaccinate as many populations as possible to the level *f*^˜^, and not vaccinate any other populations (except for perhaps one with any extra doses).

However, Duijzer et al. [12] prove that these features do not hold when there are no active infections (e.g., prior to the outbreak). In this case, *f*^¯^ = *f*^˜^ = *f*^∗^. That is, *G* is an increasing and convex function prior to the herd immunity threshold and a decreasing and concave function after the herd immunity threshold. In this case, the results of Duijzer et al. align with those of Keeling and Shattock [13]: the optimal allocation scheme is to vaccinate as many populations as possible up to the herd immunity threshold. The difference from the previously described situation is that the peak is always delayed by increasing the vaccinations in a population with no active infections. Since pre-outbreak vaccination leads to a decreased transmission rate for all cases in the population, the reduced number needed to reach the pandemic peak is always outweighed by the increased time required to infect those individuals. Duijzer et al. [14] previously demonstrated that, in this case, maximizing the herd effect is achieved when *R*_t_ = 1, i.e. at the herd immunity threshold, so this comports with that finding as well.

**References:**

[1] Bubar KM, Reinholt K, Kissler SM, Lipsitch M, Cobey S, Grad YH, et al. Model-informed COVID-19 vaccine prioritization strategies by age and serostatus. Science (1979) 2021;371:916–21. https://doi.org/10.1126/science.abe6959.

[2] Kissler SM, Tedijanto C, Goldstein E, Grad YH, Lipsitch M. Projecting the transmission dynamics of SARS-CoV-2 through the postpandemic period. Science 2020;368:860–8. https://doi.org/10.1126/science.abb5793.

[3] McMorrow M. Improving communications around vaccine breakthrough and vaccine effectiveness. 2021.

[4] Presanis AM, De Angelis D, Team3¶ TNYCSFI, Hagy A, Reed C, Riley S, et al. The Severity of Pandemic H1N1 Influenza in the United States, from April to July 2009: A Bayesian Analysis. PLoS Med 2009;6:e1000207.

[5] Lee BY, Brown ST, Korch GW, Cooley PC, Zimmerman RK, Wheaton WD, et al. A computer simulation of vaccine prioritization, allocation, and rationing during the 2009 H1N1 influenza pandemic. Vaccine 2010;28:4875–9. https://doi.org/10.1016/j.vaccine.2010.05.002.

[6] Doria-Rose N, Suthar MS, Makowski M, O’Connell S, McDermott AB, Flach B, et al. Antibody Persistence through 6 Months after the Second Dose of mRNA-1273 Vaccine for Covid-19. New England Journal of Medicine 2021;384:2259–61. https://doi.org/10.1056/NEJMc2103916.

[7] Tartof SY, Slezak JM, Fischer H, Hong V, Ackerson BK, Ranasinghe ON, et al. Effectiveness of mRNA BNT162b2 COVID-19 vaccine up to 6 months in a large integrated health system in the USA: a retrospective cohort study. The Lancet 2021;398:1407–16. https://doi.org/10.1016/S0140-6736(21)02183-8.

[8] Clarke KEN, Jones JM, Deng Y, Nycz E, Lee A, Iachan R, et al. Seroprevalence of Infection-Induced SARS-CoV-2 Antibodies — United States, September 2021–February 2022. MMWR Morb Mortal Wkly Rep 2022;71:606–8. https://doi.org/10.15585/mmwr.mm7117e3.

[9] Rosenberg ES, Tesoriero JM, Rosenthal EM, Chung R, Barranco MA, Styer LM, et al. Cumulative incidence and diagnosis of SARS-CoV-2 infection in New York. Ann Epidemiol 2020;48:23-29.e4. https://doi.org/10.1016/j.annepidem.2020.06.004.

[10] Williamson EJ, Walker AJ, Bhaskaran K, Bacon S, Bates C, Morton CE, et al. Factors associated with COVID-19-related death using OpenSAFELY. Nature 2020;584:430–6. https://doi.org/10.1038/s41586-020-2521-4.

[11] van den Driessche P. Reproduction numbers of infectious disease models. Infectious Disease Modelling 2017;2:288–303. https://doi.org/10.1016/j.idm.2017.06.002.

[12] Duijzer LE, van Jaarsveld WL, Wallinga J, Dekker R. Dose-Optimal Vaccine Allocation over Multiple Populations. Prod Oper Manag 2018;27:143–59. https://doi.org/10.1111/poms.12788.

[13] Keeling MJ, Shattock A. Optimal but unequitable prophylactic distribution of vaccine. Epidemics 2012;4:78–85. https://doi.org/10.1016/j.epidem.2012.03.001.

[14] Duijzer E, van Jaarsveld W, Wallinga J, Dekker R. The most efficient critical vaccination coverage and its equivalence with maximizing the herd effect. Mathematical Biosciences 2016;282:68–81. https://doi.org/10.1016/j.mbs.2016.09.017.
